# One variation, two regulatory modes? Novel insights into the transcriptional regulation of 1-FEH isoforms in chicory under abiotic stress

**DOI:** 10.1186/s12870-026-10378-z

**Published:** 2026-11-10

**Authors:** Jiaxin Zhong, Cornelia Walter, Gracia Stefanic, Karsten Harms, Thomas Rausch, Steffen Greiner

**Affiliations:** 1 Heidelberg University, Im Neuenheimer Feld 360, D-69120 Heidelberg, Germany; 2 Hohenheim University, Otto-Sander-Str. 5, D-70599 Stuttgart, Germany; 3 Werk Offstein, Wormser Straße 11, D-67283 Südzucker, Obrigheim/Pfalz, AG Germany; 4 Heidelberg Center for the Environment, Heidelberg University, Berliner Strasse 47, 69120 Heidelberg, Germany

**Keywords:** Chicory, 1-FEH, CiDREB, CiNAC, Cold induction, Transcriptional regulation

## Abstract

**Background:**

Chicory taproots are a major source of industrially extracted inulin. However, low temperatures at the end of the growing season cause significant inulin degradation. Inulin loss has been attributed to fructan 1-exohydrolase (1-FEH) activity, which is mediated by three isoforms, 1-FEH1, 1-FEH2a and 1-FEH2b, respectively. Previous breeding studies have indicated a prominent role for 1-FEH2b, while the relative contributions of different 1-FEH isoforms have not yet been clearly defined.

**Results:**

We demonstrate that promoters of *1-FEH1* and *1-FEH2b* (*p1-FEH1*, *p1-FEH2b*) are differentially activated by cold-inducible transcription factors CiNAC5 and CiDREB2, respectively. Furthermore, differential responses were observed for promoters of the closely related genes *1-FEH2a* and *1-FEH2b*, caused by a single-base variation in their *DRE* element core motif: While in *p1-FEH2b* the core motif *GTCG* mediates effective binding to and activation by CiDREB2, the respective sequence in *p1-FEH2a* (*TTCG*) renders this promoter insensitive to CiDREB2. Furthermore, we observed a strong co-activation of *p1-FEH2b* by CiDREB2B and CiMYB5, corroborated by direct protein-protein interaction of these transcription factors. Remarkably, all three *1-FEH* genes are also activated by high temperatures and drought, i.e., climate change-related abiotic stress factors.

**Conclusions:**

The distinct expression patterns of *1-FEH1*, *1-FEH2a*, and *1-FEH2b* under cold treatment suggest that these isoforms have specific roles in responding to cold stress. However, these genes are also likely to be involved in chicory’s response to climate change-related abiotic stress factors such as high temperatures and drought. The differential regulation of their corresponding promoters reveals distinct signaling pathways. In summary, our observations shed light on the role of inulin metabolism in chicory’s response to abiotic stress and identify potential targets for improving chicory inulin quality via targeted promoter editing.

**Supplementary Information:**

The online version contains supplementary material available at https://doi.org/10.1186/s12870-026-10378-z.

## Background

Chicory (*Cichorium intybus* L.) is a biennial plant with global scale cultivation for diverse purposes. In chicory taproots, the poly-fructan inulin (DP ranging from 10 to 30 units) serves as a storage carbohydrate, whereas fructans with low DP were found to mediate membrane protection during osmotic and freezing stress responses [1, 2]. Inulin has versatile applications in pharmaceutical and food industries, such as drug delivery, stabilizing proteins, and serving as a prebiotic dietary fiber [3]. Its low caloric value makes it suitable for food supplementation, replacing fats in products like yogurt and ice cream [4]. Application and commercial value of inulin depend on its degree of polymerization (DP). Plant growth conditions and harvest time, in particular cold-induction in late season, strongly impact on inulin quality and quantity.

Biosynthesis of inulin is initiated by 1-SST (sucrose: sucrose 1-fructosyltransferase) activity, which generates 1-kestose and glucose, while chain elongation is mediated by 1-FFT (fructan: fructan 1-fructosyltransferase) activity. Degradation of inulin is caused by the activity of 1-FEH (fructan 1-exohydrolase), which sequentially removes terminal fructose units. Chicory possesses three 1-FEH isoforms (1-FEH1, 1-FEH2a and 1-FEH2b), which are cold-inducible and thus contribute to the decrease of inulin DP in response to low temperature during late harvest season, eventually causing substantial loss of inulin quantity and quality. Remarkably, the functional loss of *1-FEH2b* has a more pronounced impact on post-harvest inulin degradation compared to the copy number variation of its close paralog *1-FEH2a*, indicating a specific role for this isoform [5]. However, the regulatory network mediating the expression of *1-FEH* genes in late autumn has remained largely unknown, requiring further investigation.

Earlier investigations have indicated that in chicory taproot enzyme activities of *f*ructan *a*ctive en*zy*mes (named FAZYs, including 1-SST, 1-FTT and 1-FEH genes) are largely correlated with the corresponding transcript levels [6], suggesting that FAZY expression is mainly regulated at the transcriptional level. Several lines of evidence support the notion that *1-FEH1*, *1-FEH2a* and *1-FEH2b* are differentially expressed in response to cold exposure [7, 8]. Thus, while under continuous cold exposure *1-FEH1* transcripts displayed persistent up-regulation, transcripts for *1-FEH2a* were only transiently induced [7]; the latter observation explaining the apparent lack of *1-FEH2a* induction when sampling was done at weekly intervals [8]. Also, while *1-FEH1* expression is already induced at 10 °C exposure, expression of *1-FEH2* isoforms kicks in only at 4 °C (unpublished data from an internal experiment). Furthermore, and consistent with the observed differential induction of *1-FEH* genes, the Ca^2+^-channel blockers Lanthanum chloride (LaCl_3_) and calmodulin antagonist N-(6-aminohexyl)-5-chloro-1-naphthalenesulfonamide repressed the cold induction of *1-FEH1* but not of *1-FEH2a* [7].

First promoter analysis of *1-FEH2a* had revealed several putative *cis*-elements for transcription factors, including DRE (dehydration responsive element: A/GCCGAC), the latter being essential for specific interactions with DREB factors (dehydration responsive element binding proteins) [9, 10]. In more recent work, several transcription factors of the R2R3-MYB family have been shown to be potential regulators of FAZY genes. While the transcription factor CiMYB17 appears to be involved in the complex regulation of synthetic and degrading FAZY genes, particularly after the exposure to abiotic stress [11], transcription factors CiMYB5 and CiMYB3 specifically activate the promoters of *1-FEH* genes by recognizing the MYB-core motifs (C/TNGTTA/G) abundantly present in the promoters of all *1-FEH* genes [12]. However, since the MYB factors tested in the promoter studies activated all three *1-FEH* gene promoters, other transcription factors are likely involved in their differential regulation.

In summary, bioinformatic and first experimental analysis had revealed that promoters of *1-FEH* genes contain a variety of *cis*-elements, indicative of complex regulatory networks. Thus, multiple binding sites are predicted for transcription factor families DREB, MYB and NAC [*No apical meristem (NAM)*,* Arabidopsis transcription activation factor (ATAF)*,* Cup-shaped cotyledon (CUC)*], respectively [13]. To further explore the differential regulation of *FEH* genes in chicory, we have in the present study (i) refined the induction kinetics of *1-FEH* genes in response to cold exposure via detailed time course analysis, (ii) identified additional transcription factors selectively activating individual *1-FEH* genes, and (iii) performed a first functional characterization of the corresponding promoter-transcription factor interactions. In particular, we have explored the involvement of *DREB subgroup 1* genes [*CiDREB1A*, *CiDREB1B;* cold-induced via ABA-independent signaling [14] and *DREB subgroup 2* genes (*CiDREB2A*,* CiDREB2B*) in the regulation of different *1-FEH* isoform genes. Moreover, the role of several CiNAC factors was addressed. For chicory *1-FEH* genes, our results provide the first experimental demonstration for isoform gene-specific responses to cold stress mediated by members of different transcription factor families. Furthermore, the induction of *1-FEH1* and *1-FEH2a/b* transcript levels in response to high temperatures and drought indicates a role in chicory’s response to climate change-related abiotic stress factors.

## Materials and methods

### Plant materials

*Cichorium intybus* L. var. Zoom seeds were sown in soil, and seedlings were grown in a greenhouse under long-day conditions (25 °C, 16 h light/8 h dark) for 4 to 6 weeks, depending on the requirements of the experiment.

### Different temperature treatments

In the first pilot experiment, 5-week-old chicory seedlings were exposed to continuous cold treatment (3 d at 4 °C followed by 3 d at room temperature) or exposed to pulsed cold treatment (three rounds of 24 h at 4 °C followed by 24 h at room temperature).

To conduct a detailed time-course analysis of cold-induced expression of 1-FEH and transcription factor genes, 4-week-old seedlings were cultivated under a 12 h light/12 h dark photoperiod. For cold treatment, chilled water was added to the growth substrate, and the seedlings were immediately transferred to a cold chamber set at 4 ± 1 °C under the same light conditions, while control plants were maintained at room temperature.

For moderate heat stress, 6-week-old seedlings were moved to an incubator at 37 °C, and root samples were collected at 0, 6, 24, 48, and 72 h after treatment. The relative humidity inside the incubator was kept at 70–80%. For severe heat stress, 6-week-old seedlings were exposed to 45 °C, and root tissues were harvested at 0, 1, 5, and 24 h post-treatment.

For each biological replicate, root tissues from two to three individual seedlings were pooled. Taproot samples were immediately frozen in liquid nitrogen and ground into a fine powder using a Mixer Mill MM 400 (Retsch GmbH, Germany), then stored at − 80 °C until further use.

### Identification and cloning of chicory *CiDREB* and *CiNAC* genes

In earlier work, Liang et al. [14] employed homologous sequences of previously characterized *CBF/DREB1* genes from other plant species as queries and identified two genes encoding *CiDREB1A* and *CiDREB1B* in chicory. In the present study, amplification of the *CiDREB1B* coding sequence using reported primers unexpectedly yielded additional isoforms, designated *CiDREB1C* and *CiDREB1D*. To identify potential CiNAC transcription factors in chicory, the conserved NAC domain from *Arabidopsis thaliana* was used as a query for nBLASTt searches against the chicory RNA database.

### Bioinformatic analysis for putative DREB/NAC binding sites

Previous analysis of chicory’s *1-FEH2a* promoter had indicated several putative *cis*-elements for transcription factors [10]. Based on the conserved binding motifs of AtDREB and AtNAC from *Arabidopsis thaliana*, the promoter regions of *1-FEH1*, *1-FEH2a*, and *1-FEH2b* were analyzed using the JASPAR database (jaspar.genereg.net) to identify *cis*-regulatory elements containing the core DRE (**D**ehydration-**R**esponsive **E**lement) or NACBS (**NAC B**inding **S**ite) motifs. (Table S1).

### Site-directed mutagenesis

Plasmid DNA carrying the target sequence was used as a template for PCR amplification with Phusion polymerase (Thermo Scientific™), following the manufacturer’s protocol. Site-specific mutations were introduced by placing the desired nucleotide substitutions in the middle of the forward primer, while positioning the 3’- end over the mutation site. The reverse primer was designed such that the 5’- ends of both primers annealed in a back-to-back orientation. Following PCR amplification, the resulting linear DNA fragments were phosphorylated using T4 Polynucleotide Kinase (Thermo Scientific™). The phosphorylated linear products were then self-ligated to generate circular plasmids using T4 DNA Ligase (Thermo Scientific™).

### Quantitative real time PCR (qRT-PCR)

Transcript abundance of target genes was quantified by qRT-PCR using a Rotor-Gene Q system (Qiagen) with 2× qPCRBIO SyGreen Mix Lo-ROX. Each 12 µl reaction contained 2 µl of cDNA template, 0.5 µl of forward primer (10 µM), 0.5 µl of reverse primer (10 µM), 6 µl of 2× qPCRBIO SyGreen Mix, and 3 µl of nuclease-free double-distilled water. Thermal cycling conditions consisted of an initial denaturation step at 95 °C for 2 min, followed by 40 cycles of 95 °C for 5 s and 62 °C for 20 s for combined annealing and extension. A melt curve analysis was subsequently performed with 1 °C increments from 63 °C to 95 °C, each held for 5 s. Relative gene expression levels were normalized against two reference genes, *Actin* and *RPL19* [12]. Primer amplification efficiencies were evaluated using standard curves generated from serial dilutions of cDNA (1:4, 1:16, 1:64, and 1:256).

### Mesophyll protoplast preparation and dual luciferase assay

The second true leaves of 3–4-week-old chicory plants were used for protoplast isolation, which was performed according to a previously published protocol from Yoo et al. [15]. Protoplast transfection was conducted based on the same method with slight modifications: for dual-luciferase assays, 10 µg of plasmid DNA (effector to reporter ratio of 6:4) was gently mixed with 100 µl of protoplasts in a 2-ml microcentrifuge tube. Subsequently, 110 µl of PEG solution (50% [w/v] PEG4000, 0.2 M mannitol, 100 mM CaCl₂) was added. The mixture was gently tapped to ensure complete and uniform mixing. The transfection mixture was incubated at room temperature for 15 min and then stopped by diluting with 440 µl of W5 solution [2 mM MES (pH 5.7), 154 mM NaCl, 125 mM CaCl_2_, 5 mM KCl] and thoroughly mixed by gentle inversion of the tube. It was then centrifuged at 100 × g for 2 min, after which the supernatant was discarded. The protoplasts were resuspended in 500 µl of WI solution [4 mM MES (pH 5.7), 0.5 M mannitol, and 20 mM KCl] and transferred to each well of a 12-well tissue culture plate. The cells were subsequently incubated at room temperature for 20 h. For measuring luciferase assay, protoplast culture was harvested at 100 × g for 2 min and further processed according to the protocol of Dual-Luciferase^®^ Reporter Assay System (Promega GmbH, Germany). Luciferase activity was measured with 96-well plate in FLUOstar Omega plate reader (BMG LABTECH GmbH, Germany).

### Electrophoretic mobility shift assay (EMSA)

Transcription factors were cloned in construct *pETG10A* and expressed in E. *coli* BL21. Recombinant protein from inclusion bodies was solubilized and refolded according to the method described in Singh et al. [16]. Labeled oligonucleotides were synthesized with a 5’- Cyanine 5 (Cy5) modification (Eurofins Genomics). The oligonucleotides were dissolved in annealing buffer (10 mM Tris, 50 mM NaCl, 1 mM EDTA, pH 7.5), and equal molar amounts were combined in a PCR tube. Annealing was performed in a thermocycler using the following program: 95 °C for 2 min, gradual cooling to 25 °C over 45 min, and a final hold at 4 °C. For binding assays, 0.2 pmol of annealed probe was incubated with 500 ng of protein in 10 µl of binding buffer [25 mM HEPES, 50 mM KCl, 0.5 mM EDTA, 0.5 mM DTT, 5% (w/v) glycerol, and 1 mg/mL BSA] for 30 min on ice. The reaction was terminated by adding 2 µl of 6× loading buffer [10 mM Tris-HCl, 0.15% (w/v) orange G, 60% (w/v) glycerol, 60 mM EDTA]. Samples were then separated on a 6% (w/v) non-denaturing polyacrylamide gel prepared in 0.5× TBE buffer. Electrophoresis was carried out at 120 V for 35 min on ice, and gel shifts were visualized using an ImageQuant LAS 4000 system. For competition experiments, unlabeled oligonucleotides were pre-incubated with the protein prior to the addition of Cy5-labeled probes.

### Yeast-two-hybrid analysis (Y2H)

Competent yeast cells (AH109) were prepared using the LiAc method. Briefly, a single healthy colony of AH109 was cultured in YPDA medium at 30 °C until the OD_600_ reached 0.15–0.3. Cells were harvested by centrifugation at 700 × g for 5 min at room temperature. The pellet was resuspended in 20 ml fresh YPDA and further incubated at 30 °C until the OD_600_ reached 0.4–0.5. Cells were then collected again by centrifugation at 700 × g for 5 min and washed with deionized water. Each pellet was resuspended in 600 µl of 1.1× TE/LiAc solution (1.1 mM EDTA, 11 mM Tris-Cl, 110 mM LiAc) and transferred into 1.5 ml microcentrifuge tubes, followed by centrifugation at high speed for 15 s. The supernatant was discarded, and the cells were resuspended in 240 µl of 1.1× TE/LiAc. The competent cells were then ready for transformation. For transformation, 50 µl of competent cells were co-transformed with 300 ng pGADT7 and 300 ng pGBKT7 constructs, together with 5 µl denatured salmon sperm DNA (D7656, Sigma). Subsequently, 500 µl PEG/LiAc solution [1 mM EDTA, 10 mM Tris-Cl, 40% (w/v) PEG3350, 100 mM LiAc] was added, and the mixture was incubated at 30 °C with gentle mixing. Then, 20 µl DMSO was added and mixed thoroughly. The tubes were heat-shocked in a 42 °C water bath for 15 min and then centrifuged at high speed for 15 s to pellet the cells. The supernatant was removed, and the pellet was resuspended in 1 ml 2× YPDA medium. Cells were centrifuged again, and the supernatant was discarded. The final pellet was resuspended in 0.9% (w/v) NaCl solution. Transformants were selected on SD/-Leu/-Trp plates at 30 °C for 3–5 days, and positive colonies were subsequently transferred to SD/-Leu/-Trp/-His/-Ade medium for 3–5 days. Positive protein–protein interactions were identified based on colony growth. For further confirmation, 4 mM X-α-Gal (Merck, 16555) was applied, and positive colonies exhibited blue coloration.

### Accession numbers

Sequence data in this study can be found in GenBank under the following accession numbers: *1-FEH1* (KY385878), *1-FEH2a* (AY323935.1), *1-FEH2b* (KY385879).

### Statistical analysis

Dual luciferase assay experiments were conducted with three biological replicates and three technical replicates. Data were analyzed using Statistics Kingdom. Differences among multiple transcription factors tested on the same promoter were assessed by one-way ANOVA, followed by Tukey’s post hoc test for multiple comparisons. Comparisons between a single transcription factor and the empty vector control were evaluated using Student’s t-test. Results are presented as mean ± SD, and differences were considered significant at *p* ≤ 0.05.

## Results

### Identification of *CiDREB* and *CiNAC* genes in chicory

Following the strategy as used for the previously characterized CiMYB transcription factors R2R3MYB family [12], we have identified several members of two additional transcription factor families, i.e. DREB factors and NAC factors from chicory. As for both transcription factor families, bioinformatics analysis predicted multiple putative binding sites in the promoters of *1-FEH* genes (Supplementary Table S1), their possible involvement in abiotic stress responses was explored.

Using LsDREB2A from *Lactuca sativa* as a query sequence, a tBLASTn search in the chicory database identified two DREB2 homologs, which were designated CiDREB2A and CiDREB2B. In addition, primers originally designed to amplify the CiDREB1B coding sequence [14] unexpectedly yielded additional closely related isoforms, named CiDREB1C and CiDREB1D, respectively. Multiple sequence alignment of CiDREB1 and CiDREB2 proteins revealed a highly conserved AP2 domain comprising 57 amino acids (Supplementary Fig. S1A). To further investigate their potential functions and evolutionary relationships, the amino acid sequences of CiDREBs were compared with *Arabidopsis thaliana* DREB proteins using maximum likelihood phylogenetic analysis. Within the subgroup 1 family, CiDREB1A, CiDREB1C, and CiDREB1D clustered with the DWARF AND DELAYED FLOWERING proteins AtDDF1 (AT1G12610.1) and AtDDF2 (AT1G63030.1). In contrast, CiDREB2A and CiDREB2B were grouped into the subgroup 2 family (Supplementary Fig. S1B). Using the same strategy, we identified and cloned eight *CiNAC* genes, named *CiNAC1-8*. Alignment of CiNAC protein sequences revealed the bipartite conserved NAC domain (Supplementary Fig. S2A).

### Differential induction kinetics of *1-FEH* genes after cold exposure

In a pilot experiment, induction kinetics of *1-FEH* transcripts were monitored in taproots of 5-week-old chicory seedlings using two scenarios, i.e. either by continuous exposure of plants to 4 °C for 3 days followed by 3 days at room temperature (RT), or by three sequential exposures of 24 h 4 °C/24 h RT (pulsed exposure) alternatively. In addition to confirming the previously reported differential expression profile for *1-FEH1* (continuous increase after cold exposure) versus *1-FEH2a* (transient increase after cold exposure) [7], the results for *1-FEH2b* revealed a similar pattern as for *1-FEH2a* (Supplementary Fig. 3). Interestingly, in both scenarios, transcripts for all three *1-FEH* genes sharply declined after transfer to RT, indicating rapid transcript degradation. Furthermore, for *1-FEH1* pulsed cold exposure indicated similar upregulation after each cold treatment, whereas for 1-FEH2a and 1-FEH2b transcript levels appeared to decline.

### Transcription factors co-expressed with *1-FEH* target genes in response to low temperature

To compare expression profiles for *1-FEH* genes and transcription factor genes at high temporal resolution, we have performed a detailed time course expression analysis over a 48-h period in taproots of 4-week-old chicory seedlings, monitoring transcript levels of *1-FEH* isoforms and *DREB/NAC* transcription factor candidates by qPCR. At this developmental stage, taproot development and inulin accumulation have already started. Plants were cultivated under a 12 h light/12 h dark regime. Control plants were grown at 25 °C, whereas for cold treatment plants were transferred to 4 °C.

**Fig. 1:**
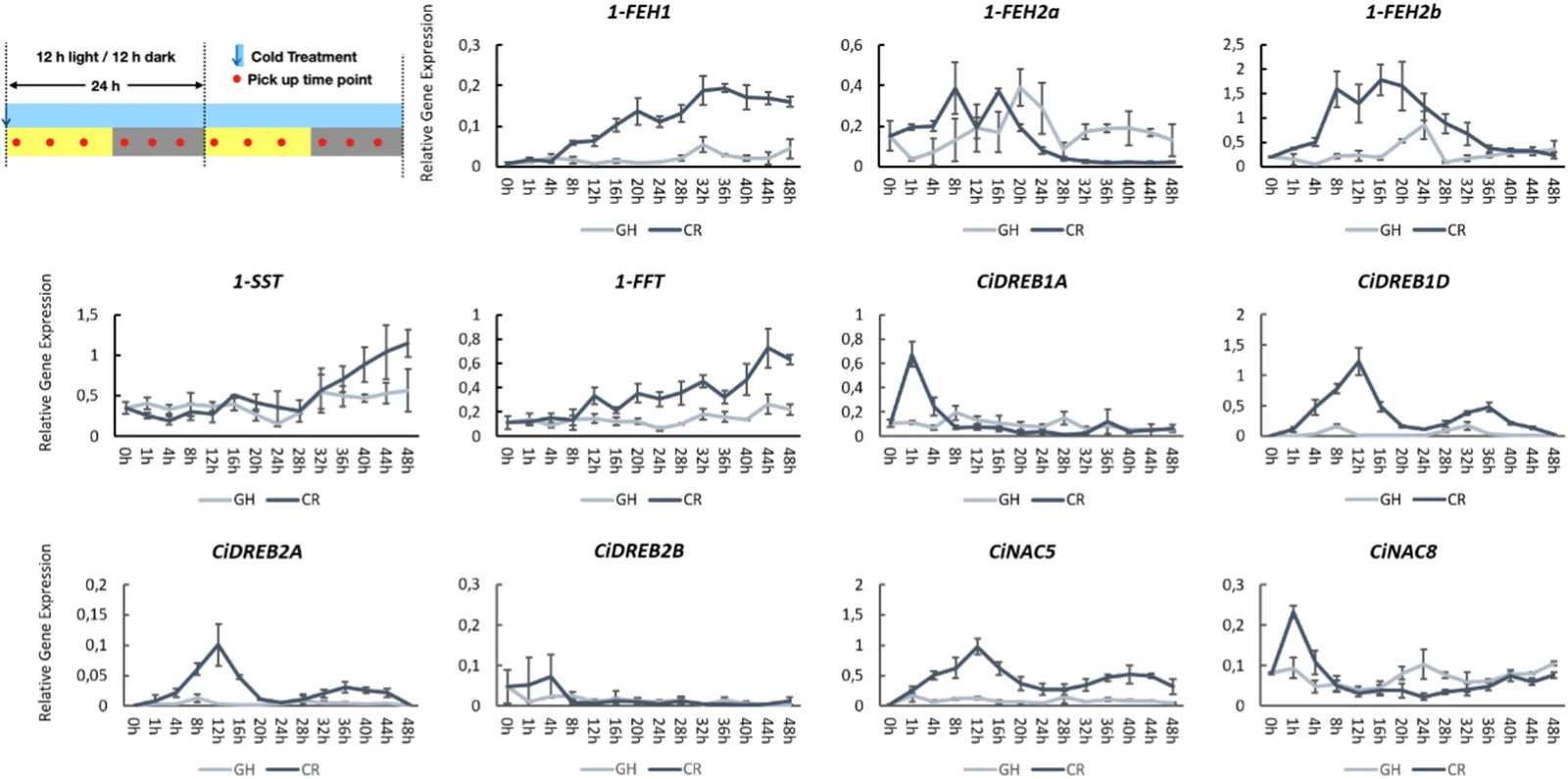
Effect of cold treatment on transcript levels of FAZY genes and transcription factors in taproots of 4-week-old chicory seedlings. Taproot samples were collected every 4 h after the onset of treatment. Diurnal variation under a 12 h light/12 h dark photoperiod is indicated in yellow and gray, respectively. Seedlings were transferred to a cold chamber (CR, 4 ± 1 °C), while control plants were maintained in a greenhouse (GH, 25 ± 2 °C). Transcript abundance was quantified by qRT-PCR and normalized to two reference genes, *Actin* and *RPL19*. Relative gene expression levels were calculated using the ΔCt method. Error bars indicate standard deviation (*n* = 3–4)

The results revealed that induction of *1-FEH1* transcripts started only after 8 h of cold treatment but then increased gradually over time, reaching a plateau after 32 h (Fig. 1). In marked contrast, *1-FEH2b* transcripts were rapidly induced, reaching a transitory plateau after 8 h with subsequent decline starting at 20 h, and reaching base level after about 36 h (Fig. 1). In comparison, expression of *1-FEH2a* transcripts upon cold treatment was only slightly and transiently induced, but the overall induction was lower and limited to the first 8 h (Fig. 1). Interestingly, expression of *1-FFT* transcripts was also moderately increased upon cold treatment, following a similar pattern as compared with *1-FEH1* transcripts, while *1-SST* transcripts displayed no significant cold induction or diurnal fluctuation (Fig. 1).

In the same experimental setting, we also monitored the expression of the newly identified DREB and NAC transcription factors. Cold induction of *CiDREB1A* transcripts reached an early and sharp peak, followed by a rapid decline to reach again base level after about 8 h, whereas transient induction of *CiDREB1D* transcripts started later, reaching peak level only after 12 h (Fig. 1). Among the two members of DREB family subgroup 2, *CiDREB2A* and *CiDREB2B*, the former showed an induction pattern similar to *CiDREB1D*, whereas *CiDREB2B* showed only a very modest and transient induction, similar to *CiDREB1A*, but with higher variability (Fig. 1). Among several CiNAC transcription factor candidates, only *CiNAC5* and *CiNAC8* transcripts displayed transient cold induction, the former peaking at 12 h, the latter at 1 h after onset of cold treatment (Fig. 1).

In summary, the observed distinct expression profiles exhibited by *1-FEH1*, *1-FEH2a*, and *1-FEH2b*, along with (i) the identification of cold-responsive DREB and NAC transcription factors, and (ii) the prediction of corresponding binding sites in the promoters of *1-FEH* genes (see Table S1), led to the hypothesis that the distinct cold response patterns of *1-FEH* isoform genes are due to their regulation by different transcription factor combinations. Consequently, the responses of *1-FEH* gene promoters to the newly identified DREB and NAC factors were explored in detail.

### CiNAC5 strongly and selectively activates the promoter of *1-FEH1*

**Fig. 2:**
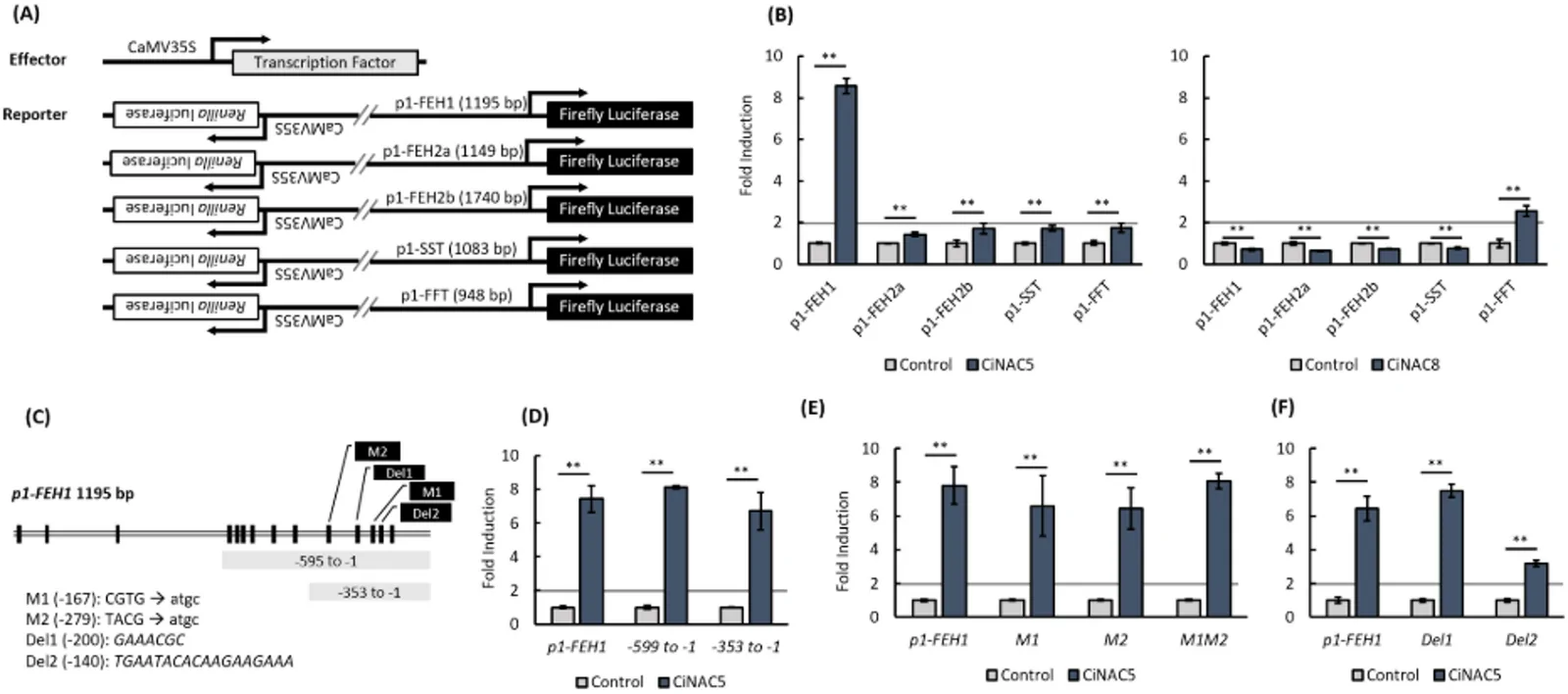
CiNAC5 selectively activates the promoter of *1-FEH1*. **A** Schematic representation of reporter constructs in which the 5’ UTR/promoter regions of *1-FEH1*, *1-FEH2a*,* 1-FEH2b*, *1-SST*, and *1-FFT* were fused upstream of a firefly luciferase gene. Each FAZY promoter-driven reporter was co-expressed with either CiNAC5 or CiNAC8 effector constructs, or with an empty vector control. The *Renilla* luciferase construct was included for normalization. **B** Relative promoter activities of FAZY genes (*1-FEH1*, *1-FEH2a*, *1-FEH2b*, *1-SST*, and *1-FFT*) in the presence of CiNAC5 or CiNAC8 compared with the empty vector control. **C** Schematic overview of *1-FEH1* promoter fragments, indicating the positions of putative NAC binding sites. Two promoter fragments (595 bp and 353 bp) were fused upstream of a firefly luciferase reporter. Mutations (M) and deletions (Del) of NACBS are indicated. **D** Promoter activities of *1-FEH1* deletion constructs in the presence of CiNAC5. **E** Promoter activity of *1-FEH1* containing mutated NAC binding sites in the presence of CiNAC5. **F** Promoter activity of *1-FEH1* with deleted NAC binding sites in the presence of CiNAC5. Basal promoter activities are shown as relative LUC activity (Firefly/Renilla). Data represent means ± SD of three technical replicates. Statistical significance was determined using Student’s t-test (* *p* ≤ 0.05, ** *p* ≤ 0.01). Results were consistent across two independent experiments

To investigate the potential regulatory influence of CiNACs on promoter activation of *1-FEH* genes, we performed a dual luciferase assay using a well-established homologous protoplast system. Promoter sequences of approximately 1 kb length from all *1-FEH* genes were cloned into the reporter construct and co-transfected with transcription factors expressed under control of the robust CaMV35S promoter (Fig. 2A). To exclude minor or random effects, a 2-fold increase in promoter activity was set as an arbitrary threshold. The results revealed that CiNAC5 selectively activated the promoter of *1-FEH1*, inducing it by 8- to 9-fold. While CiNAC5 or CiNAC8 did not show significant activation of *1-FEH2a* and *1-FEH2b* promoters (Fig. 2B).

Based on the strong and selective induction of the *1-FEH1* promoter by CiNAC5, a promoter deletion analysis was performed to narrow down the location of the NAC binding site. Three different deletions of *p1-FEH1* fragments were cloned into the reporter constructs, each containing varying numbers of putative NAC binding sites (Fig. 2C). The results revealed that none of these deletions suppressed promoter induction, suggesting that the region from − 353 to ATG contains NAC binding site(s) that are sufficient to convey full activation by CiNAC5 (Fig. 2D). Within this region, four putative NAC binding sites were further analyzed via mutation or deletion. Surprisingly, single mutations in the core motifs of two NAC binding sites (CGTC, located − 167 upstream of ATG; TACG, located − 279 upstream of ATG) and even their double mutation did not affect activation of *p1-FEH1* by CiNAC5 (Fig. 2E). Similarly, complete deletion of NAC binding sites − 200 upstream of ATG did not affect promoter activation. However, promoter deletion down to -140 upstream of ATG strongly decreased CiNAC5-mediated activation of *p1-FEH1* (Fig. 2F).

### CiDREB1 and CiDREB2 selectively activate the promoter of *1-FEH2b* but not of *1-FEH2a*

**Fig. 3:**
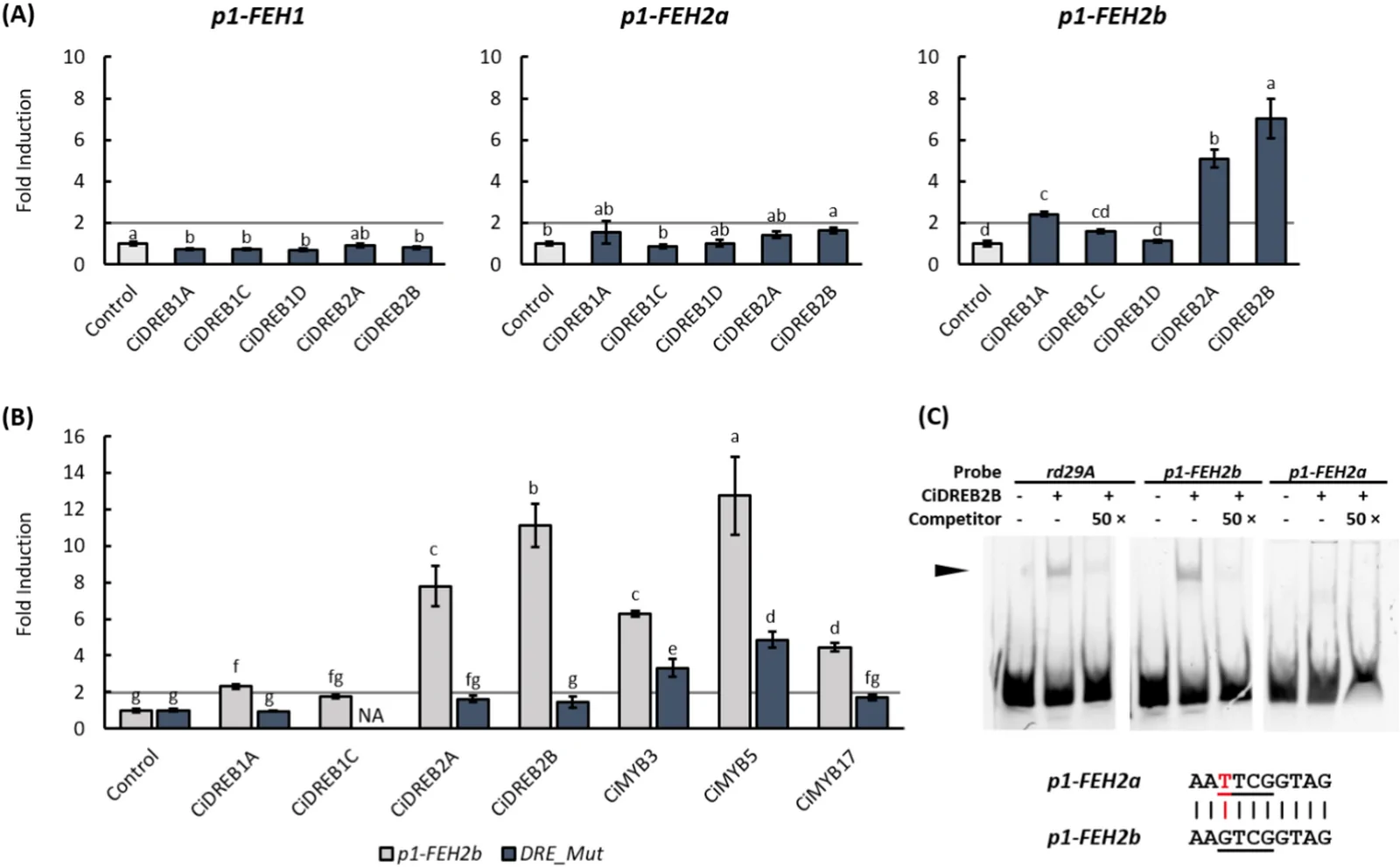
Effects of CiDREB1 and CiDREB2 on the promoters of *1-FEH1*, *1-FEH2a* and *1-FEH2b*. For reporter construct, the 5’ UTR/promoter regions of *1-FEH1*, *1-FEH2a* and *1-FEH2b* were fused upstream of a firefly luciferase gene. Each promoter-driven reporter was co-expressed with either CiDREB1 or CiDREB2 effector constructs, or with an empty vector control. The *Renilla* luciferase reporter was included in all constructs for normalization. **A** Relative induction of *1-FEH* promoter activities in the presence of CiDREB1 or CiDREB2 compared with the empty vector control. **B** Effect of *DRE* motif mutation (DRE_Mut; AAtTCtGTAG at position − 138) on *1-FEH2b* promoter activity. Basal promoter activity is expressed as relative LUC activity (Firefly/Renilla). Data represent means ± SD of three technical replicates. Statistical significance was assessed using one-way ANOVA (*p* ≤ 0.05). Results were confirmed in two independent experiments. **C** Electrophoretic mobility shift assays (EMSA) showing binding of CiDREB2B protein to promoter fragments of *1-FEH2a* and *1-FEH2b*, with *rd29A* used as a positive control. A full-length 6×His-tagged CiDREB2B protein was used for binding assays. DNA–protein complexes are indicated by black arrowheads. Cy5-labeled 40-mer promoter fragments (0.2 pmol) were used for detection, and 50-fold excess unlabeled probes were included as competitors

To explore the potential regulatory role of CiDREBs for activating *1-FEH* gene promoters, we used the same approach as described above. A first functional analysis indicated that DREB factors CiDREB1A/1 C did not show significant activation of *p1-FEH2b*, whereas CiDREB2A/2B caused pronounced activation (6- to 8- fold) (Fig. 3A). Intriguingly, despite sharing a high sequence similarity with *p1-FEH2b*, *p1-FEH2a* did not respond to either CiDREB1 or CiDREB2 factors (Fig. 3A). Likewise, *p1-FEH1* was unaffected by CiDREB1 and CiDREB2 factors.

To elucidate the cause for selective activation of *p1-FEH2b* but not of *p1-FEH2a*, we screened both promoters for the presence of putative DRE element (see Table S1). This led to the identification of a DRE element on *p1-FEH2a* (AA**TTCG**GTAG, located − 136 upstream of ATG), which displays a single base pair variation in its core motif compared to *p1-FEH2b* (AA**GTCG**GTAG, located − 138 upstream of ATG). To confirm the possible role of this putative DRE sequence for the activation of *p1-FEH2b* by CiDREB factors, we generated a DRE mutation (AA**tTCt**GTAG at site − 138) in *p1-FEH2b*. As expected, deletion or mutation of the *DRE* element in the *1-FEH2b* promoter abolished its activation by CiDREB2A and CiDREB2B, respectively (Fig. 3B and Supplementary Fig. S4A).

Subsequent analysis via electrophoretic mobility shift assay (EMSA) revealed that recombinant CiDREB2B protein specifically interacted with *p1-FEH2b* through recognition of this DRE element, whereas it did not bind to *p1-FEH2a* presumably due to the presence of a natural mutation in its DRE core-motif (Fig. 3C). Consistent observations were also obtained with other members, including CiDREB1A, CiDREB1C, CiDREB1D, and CiDREB2A, as demonstrated in Supplementary Fig. S3. Together, these outcomes substantiate the specific and differential binding abilities of CiDREB transcription factors to the promoters of *1-FEH2a* and *1-FEH2b*, elucidating their distinct regulatory features in chicory.

### CiMYB5/3 and CiDREB2B synergistically co-activate *p1-FEH2b*

**Fig. 4:**
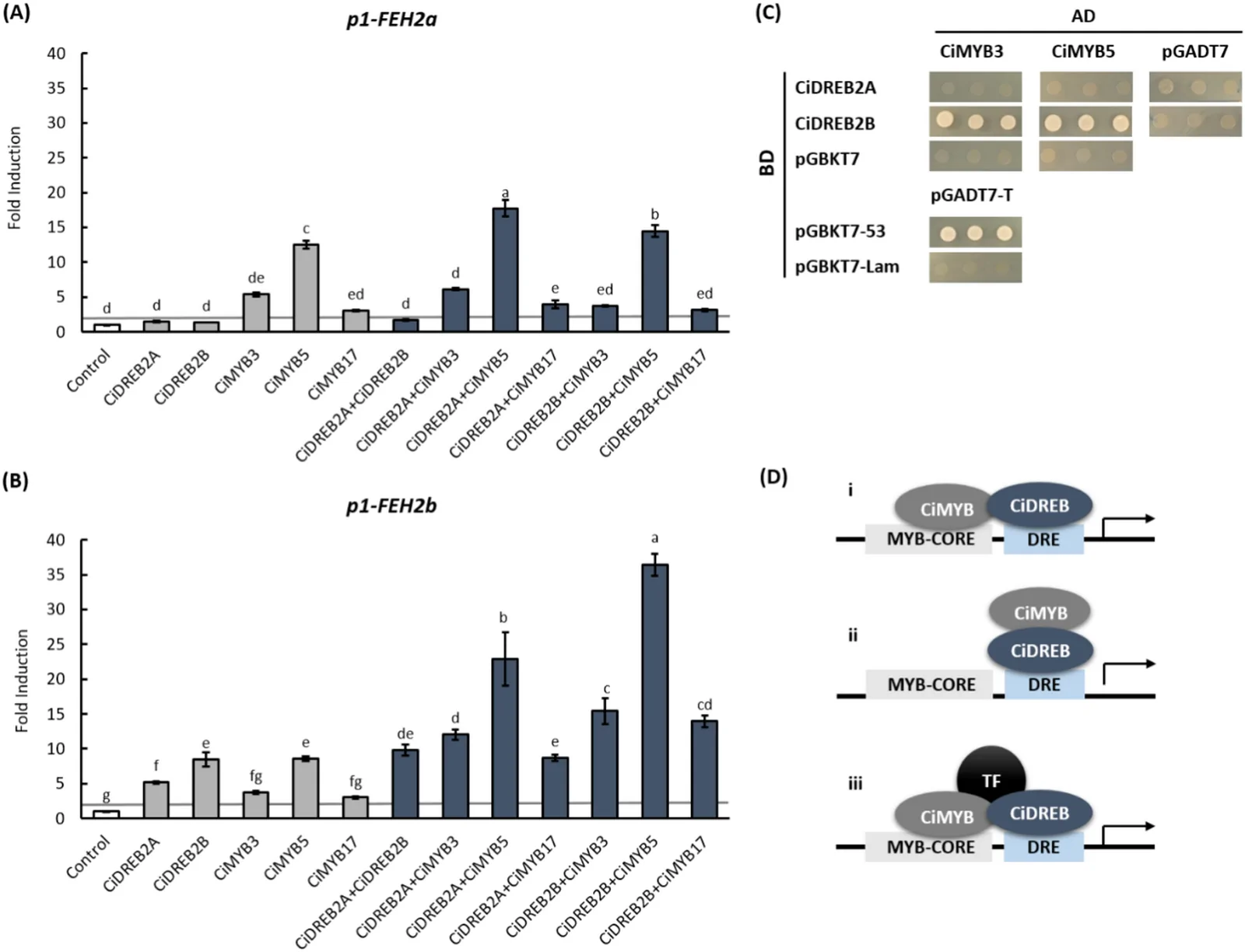
Effect of CiDREB2 and CiMYBs on promoter activities of *1-FEH2a* and *1-FEH2b*. **A** Relative promoter activity of *1-FEH2a* in the presence of CiDREB and/or CiMYB proteins compared with the empty vector control. **B** Relative promoter activity of *1-FEH2b* in the presence of CiDREB and/or CiMYB proteins, showing combined effects of both transcription factors compared with single-factor expression and the empty vector control. Basal promoter activity is expressed as relative LUC activity (Firefly/*Renilla*). Data represent means ± SD of three technical replicates. Statistical significance was determined using one-way ANOVA (*p* ≤ 0.05), with results confirmed in two independent experiments. **C** Protein–protein interaction analysis between CiDREB and CiMYB proteins using yeast two-hybrid assays, showing growth on selective media and reporter gene activation. Positive and negative controls are included for comparison. **D** Schematic model illustrating the hypothetical regulatory interaction between CiMYB5 and CiDREB2B in controlling *1-FEH2b* promoter activity

To examine the relationship between CiMYB5/3 and CiDREB2B, co-transfection assays were performed using both transcription factors. When expressed individually, CiDREB2B and CiMYB5 each induced *p1-FEH2b* activity by approximately 8- to 9-fold. In contrast, co-expression of CiDREB2B and CiMYB5 resulted in a markedly stronger activation of the *p1-FEH2b* (approximately 36-fold), suggesting a synergistic effect between the two factors (Fig. 4B). As expected, CiMYB3- and CiMYB5-mediated activation of the *1-FEH2a* promoter was not influenced by co-transfection with CiDREB proteins (Fig. 4A).

Subsequent bioinformatic analysis predicted a MYB-binding core motif (TAACTA) in *p1-FEH2b*, located − 148 base upstream from the ATG start codon of *1-FEH2b*, i.e. in close vicinity to the DRE sequence. Motivated by the observed co-activation of *p1-FEH2b* and the close vicinity of the DREB and MYB core motifs, we conducted a yeast-two-hybrid assay to investigate whether these two transcription factor proteins interact with each other. As shown in Fig. 4C, CiDREB2B exhibited protein–protein interactions with both CiMYB3 and CiMYB5, whereas CiDREB2A did not show such interactions. The same observation was also confirmed by the luciferase complementation assay (Supplementary Fig. S8).

While CiDREB2B has been experimentally confirmed to bind to the promoter of *1-FEH2b* via the DRE element (Fig. 3C), binding of CiMYB5 to the close-by predicted MYB core motif could not yet be demonstrated as attempts to express CiMYB5 protein in prokaryotic systems failed. Based on the demonstrated protein-protein interaction, we hypothesize that both factors, CiDREB2B and CiMYB5/3, attach to their adjacent binding sites, causing cooperative promoter activation, however, additional protein factors could be involved (Fig. 4D). Further investigations are required to elucidate the precise mechanism by which CiMYB5/3 and CiDREB2B co-activate the promoter of *1-FEH2b*. Elucidating this interaction will provide valuable insights into the regulatory network governing the transcriptional activation of *1-FEH2b* in chicory.

### High temperatures and water deficiency affect the expression of genes related to fructan metabolism

The aforementioned results showed that CiDREB2B interacts with CiMYB3/5, leading to cooperative activation of *p1-FEH2b*, however, as in young chicory seedlings expression of *CiDREB2B* transcripts exhibited only a short and transient response to cold treatment (Fig. 1), it remains to be confirmed whether this activation mechanism plays a role in the cold response. As in the model plant *Arabidopsis thaliana*, the DREB subgroup 2 transcription factor is known to respond to drought and heat stress, we have also explored whether expression of *CiDREB2* and *1-FEH* genes is affected by these stress factors.

**Fig. 5:**
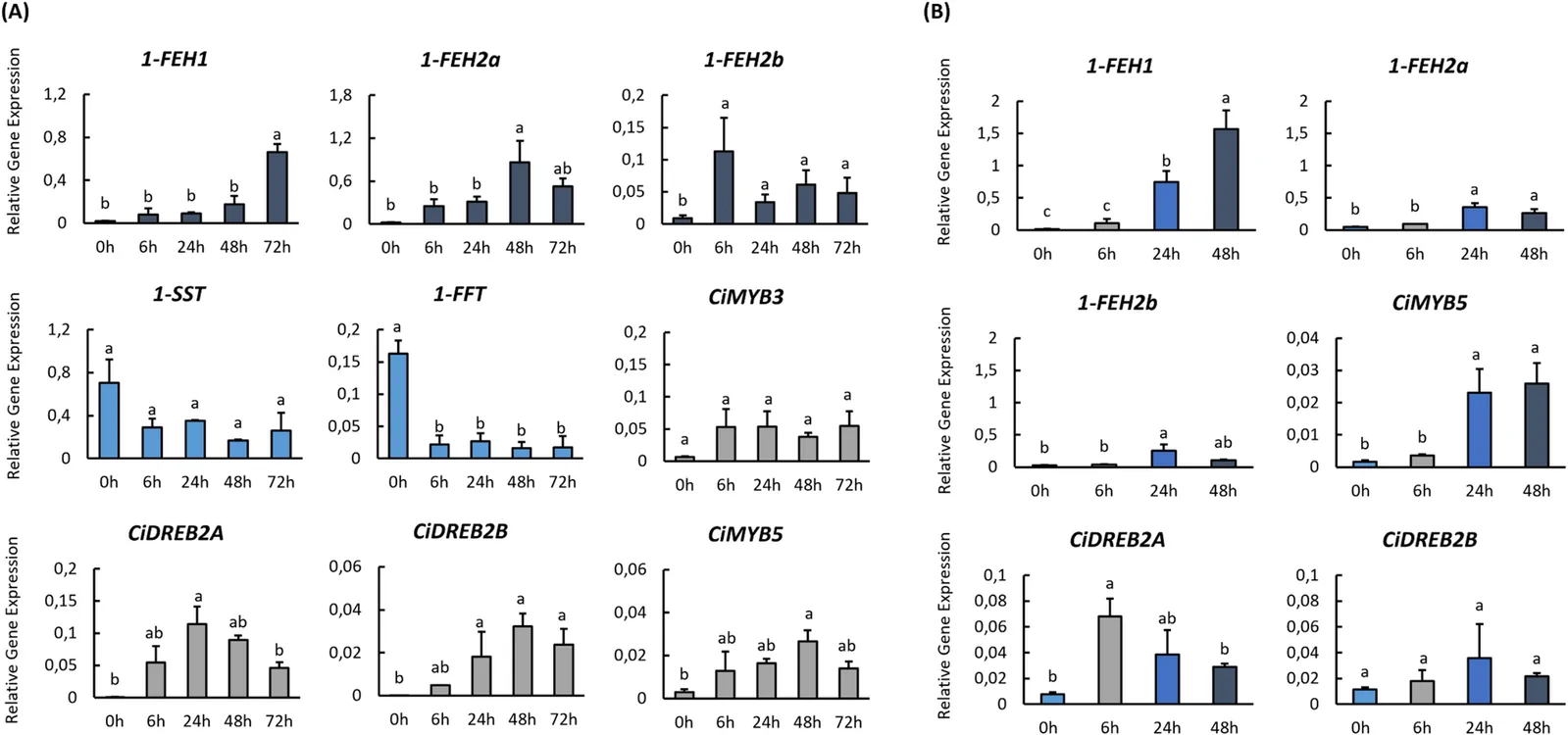
Impact of heat treatment and water deficiency on transcript levels of FAZY and transcription factor genes in taproots of 6-week-old chicory seedlings. **A** Seedlings were exposed to heat treatment at 37 ± 1 °C, and taproot samples were collected at the indicated time points after the onset of treatment. **B** For water deficiency treatment, seeds were germinated on water-soaked sponges until the first true leaves developed, after which plants were transferred to hydroponic culture with Hoagland solution [17]. Six weeks after germination, plants were removed from the nutrient solution and roots were drained to induce water deficiency, with taproot samples collected at the indicated time points. Transcript levels were quantified by qRT-PCR and normalized to two reference genes, *Actin* and *RPL19* [12]. Relative gene expression was calculated using the ΔCt method. Data represent means ± SD (*n* = 3), and statistical significance was evaluated using one-way ANOVA (*p* ≤ 0.05)

Remarkably, expression of all three isoforms of *1-FEH* was induced in response to 37 °C exposure (Fig. 5A). However, while *1-FEH1* and *1-FEH2a* transcripts exhibited significant up-regulation only after 48 h and 72 h, respectively, of heat treatment, a strong and consistent increase of *1-FEH2b* transcripts was already observed after 6 h of heat treatment. In marked contrast, the expression of fructan biosynthesis genes, *1-SST* and *1-FFT*, was rapidly and consistently repressed (Fig. 5A). Similar trends were observed even at a higher temperature (45 °C; Supplementary Fig. S5). Not unexpected, *CiDREB2A* and *CiDREB2B* transcripts were up-regulated as early as 6 h after heat treatment (37 °C) (Fig. 5A). Conversely, the specific regulation of *p1-FEH1* by CiNAC5 as suggested for cold treatment apparently plays no role during the heat response as under this condition expression of *1-FEH1* (Fig. 5A) and *CiNAC5* (Supplementary Fig. S6) show opposite patterns.

In response to water deficiency, the expression of *1-FEH1* was strongly induced, including a significant further increase between 24 h and 48 h of stress exposure (Fig. 5B), whereas the impact on expression of *1-FEH2a* and *1-FEH2b* was less pronounced. Among the transcription factors, *CiDREB2A* displayed an early induction after 6 h of drought treatment, followed by a gradual decline, whereas *CiMYB5* expression showed a delayed induction after 24 h of water deficiency. Water deficiency had opposite effects on the expression of *1-FEH1* (Fig. 5B) and *CiNAC5* (Supplementary Fig. S6).

## Discussion

### Identification of DREB and NAC transcription factors in chicory

Phylogenetic analysis classified CiDREB1A, CiDREB1C, and CiDREB1D within the DREB subgroup 1 family. These genes show similarity to the DWARF AND DELAYED FLOWERING genes *AtDDF1* and *AtDDF2* in *Arabidopsis thaliana*, whose overexpression has been shown to enhance tolerance to high salinity [18]. CiDREB2A and CiDREB2B clustered within the DREB subgroup 2 family (Supplementary Fig. S1B), which also includes AtDREB2A and AtDREB2B, transcription factors known to regulate drought-responsive gene expression [19]. These phylogenetic relationships suggest that CiDREB genes may play important roles in mediating abiotic stress responses in chicory.

Evolutionary comparison of chicory NAC transcription factors with NAC proteins from *Arabidopsis thaliana* revealed a close relationship between CiNAC5 and ANAC017 (AT1G3190.1, Supplementary Fig. S2B). ANAC017 has been reported to function in mitochondrial retrograde signaling and to regulate mitochondrial respiratory capacity during reductive stress [20]. This similarity suggests that CiNAC5 may also participate in stress-related signaling pathways.

### Distinct transcriptional activation pathways of *1-FEH* genes in response to cold exposure

Under cold treatment, the expression of *1-FEH2a* and *1-FEH2b* was strongly upregulated, whereas *1-FEH1* transcript levels increased gradually over time (Fig. 1). This suggests that 1-FEH1 and 1-FEH2 may play distinct roles in response to short-term temperature fluctuations and long-term seasonal temperature changes. Cold treatment moderately increased the abundance of *1-FFT* transcripts, following a pattern similar to that observed for *1-FEH1*. This observation may indicate a dual role of 1-FFT in both fructan elongation and degradation during cold exposure. The transcription factor genes *CiDREB1D*, *CiDREB2A*, and *CiNAC5* displayed a bimodal expression pattern (Fig. 1), with transcript levels increasing during the light phase and declining during the dark phase. This pattern suggests that cold-induced transcriptional regulation may interact with light-dependent or circadian regulatory mechanisms.

Promoter deletion analysis revealed that removal of sequences upstream of − 140 relative to the ATG start codon strongly reduced CiNAC5-mediated activation of the *1-FEH1* promoter (Fig. 2), indicating that the relevant NAC-binding site(s) are located close to the transcription start site. Furthermore, mutation of a DRE element in the *1-FEH2b* promoter disrupted activation by CiDREB factors, highlighting its crucial role in regulating *1-FEH2b* transcription. Interestingly, this mutation also reduced activation by CiMYB5 and CiMYB3, suggesting that CiDREB factors are required for full CiMYB-mediated activation and indicating a functional interaction between CiMYB and CiDREB transcription factors.

In the dual luciferase assay, co-transfection of CiDREB2B and CiMYB5 resulted in higher activation of the *1-FEH2b* promoter compared to their individual transfections (Fig. 4B). Together with the yeast two-hybrid results, which indicate an interaction between CiDREB2B and CiMYB5 (Fig. 4C), these findings suggest a context-dependent regulatory mechanism in which CiDREB2B and CiMYB5 proteins cooperatively and synergistically activate *1-FEH2b* expression.

### Expression of *1-FEH* genes in response to climate change-related abiotic stress

Heat stress has been reported to induce early bolting and flowering in chicory [21], suggesting a link between temperature and developmental transitions. In this context, heat-induced expression of *1-FEH* genes may function similarly to vernalization by promoting inulin degradation and thereby supplying energy for flowering. Consistent with this hypothesis, *CiDREB2A* and *CiDREB2B* transcripts were upregulated as early as 6 h after heat treatment (37 °C), indicating a rapid transcriptional response. This observation suggests that co-activation of the *1-FEH2b* promoter by CiDREB2B and CiMYB5 may also contribute to heat-induced *1-FEH2b* expression. Oligo-fructans have been proposed to stabilize cellular membranes under abiotic stresses such as drought [22]. Under water deficiency, we observed the upregulation of *1-FEH1*, while *CiNAC5* exhibited opposite expression patterns, suggesting that CiNAC5 regulates *1-FEH1* induction in response to cold conditions rather than during drought stress.

**Fig. 6:**
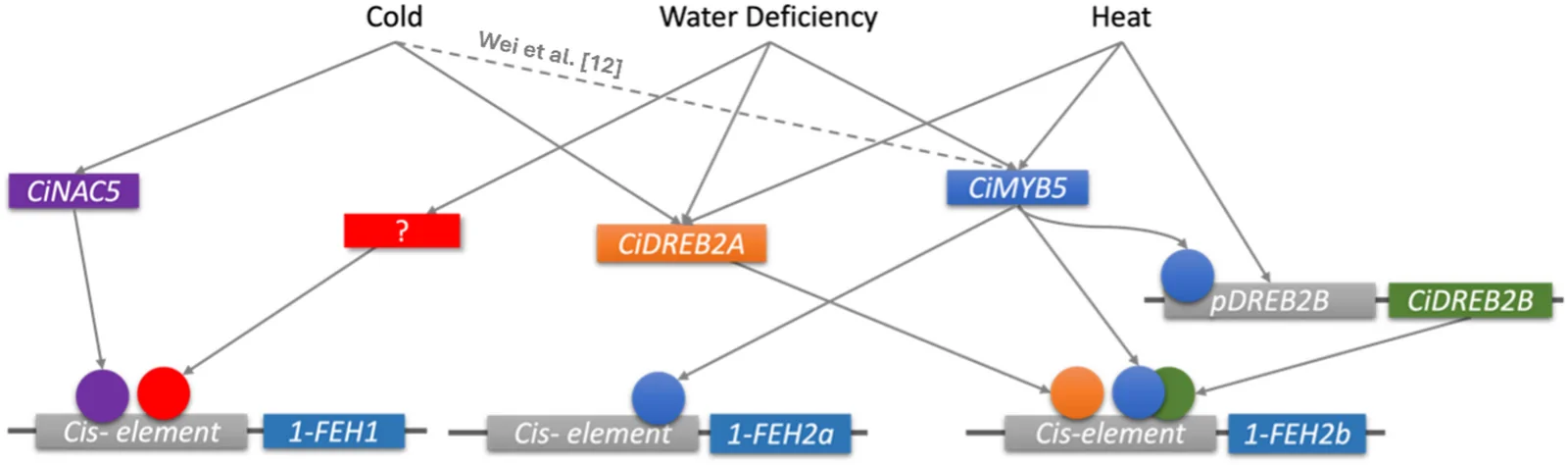
Proposed transcriptional regulatory network for control of chicory *1-FEH* genes exposed to abiotic stress. The solid line with arrows indicates positive regulatory relationships validated in this study. The dashed line represents the cold-induced expression of CiMYB5, which was previously reported by Wei et al. [12] but not experimentally confirmed in the present work

## Conclusion

In summary, this study offers new insight into the transcriptional regulation of chicory’s fructan 1-exohydrolases isoforms *1-FEH1*,* 1-FEH2a*, and *1-FEH2b*, respectively, under various stress regimes, i.e. cold, heat, and drought (Fig. 6). The results indicate specific activation mechanisms for expression of the different 1-FEH isoforms: While the presented evidence strongly suggests that CiNAC5 regulates *1-FEH1* expression under cold exposure, CiDREB2A may mediate responses to multiple abiotic stresses, specifically regulating *1-FEH2b*. Furthermore, the differential regulation of *1-FEH2a* and *1-FEH2b* by several CiDREB factors could be attributed to a natural mutation in the DRE core motif of *p1-FEH2a.* Thus, the resulting absence of *p1-FEH2a* activation by CiDREB factors may offer a mechanistic understanding for the previous observation that loss of function of *1-FEH2b* has a greater impact on post-harvest inulin degradation as compared to copy number variation of *1-FEH2a* [5]. The synergistic co-activation of *p1-FEH2b* by CiDREB2B and CiMYB5/3 via protein-protein interaction may represent a novel regulatory principle and deserve further study.

We conclude that in agreement with earlier reports, the different time courses for cold induction of transcripts for *1-FEH1* (continuous) and *1-FEH2a/b* (transient), respectively, represent robust reproducible patterns. Different physiological functions of individual 1-FEH isoforms during plant development are orchestrated via distinct transcriptional controls. Whether - and if how - enzyme properties of different chicory 1-FEH isoforms also include functional specialization remains an open question. Interestingly, a report on two distinct FEH isoforms of Edible Burdock (*Arctium lappa* L.) revealed differential activities towards long and short chain fructans, respectively [23]. Last but not least, the presented results are not only relevant for our understanding of late harvest-related inulin degradation but also demonstrate for the first time that *1-FEH* genes may be involved in the plant’s response to climate change-related abiotic stress.

This work is based on the author’s Dissertation (Jiaxin Zhong, 2023) [24].

## Supplementary Information

Below is the link to the electronic supplementary material.

Supplementary Material 1

Supplementary Material 2

## Data Availability

All data supporting the findings of this study are available within the paper and its supplementary information.

## Abbreviations

1-FEH: Fructan 1-exohydrolase
1-FFT: Fructan: fructan 1-fructosyltransferase
1-SST: Sucrose: sucrose 1-fructosyltransferase
DRE: Dehydration Response Element
DREB: Dehydration Responsive Element-Binding protein
FAZY: Fructan active enzyme
NAC: No apical meristem (NAM), Arabidopsis transcription activation factor (ATAF), Cup-shaped cotyledon (CUC)
NACBS: NAC Binding Site
TF: Transcription factor

## References

[1] Valluru R, Van den Ende W. Plant fructans in stress environments: emerging concepts and future prospects. J Exp Bot. 2008;59(11):2905–16. 10.1093/jxb/ern164. 18603617

[2] Benkeblia N. Insights on fructans and resistance of plants to drought stress. Front Sustainable Food Syst. 2022;6:827758. 10.3389/fsufs.2022.827758.

[3] Mensink MA, Frijlink HW, van der Voort Maarschalk K, Hinrichs W. Inulin, a flexible oligosaccharide. II: Review of its pharmaceutical applications. Carbohydr Polym. 2015;134:418–28. 10.1016/j.carbpol.2015.08.022. 26428143

[4] Balthazar CF, Silva H, Esmerino EA, Rocha RS, Moraes J, Carmo M, Azevedo L, Camps I, Abud Y, Sant’Anna C, Franco RM, Freitas M, Silva MC, Raices R, Escher GB, Granato D, Ranadheera CS, Nazarro F, Cruz AG. The addition of inulin and Lactobacillus casei 01 in sheep milk ice cream. Food Chem. 2018;246:464–72. 10.1016/j.foodchem.2017.12.002. 29291874

[5] Dauchot N, Raulier P, Maudoux O, Notté C, Draye X, Van Cutsem P. Loss of function of *1-FEH IIb* has more impact on post-harvest inulin degradation in *Cichorium intybus* than copy number variation of its close paralog *1-FEH IIa*. Front Plant Sci. 2015;6:455. 10.3389/fpls.2015.00455. PMC4477480 26157446

[6] Van Laere A, Van den Ende W. Inulin metabolism in dicots: Chicory as a model system. Plant Cell Environ. 2002;25(6):803–13. 10.1046/j.1365-3040.2002.00865.x.

[7] Kusch U, Greiner S, Steininger H, Meyer AD, Corbière-Divialle H, Harms K, Rausch T. Dissecting the regulation of fructan metabolism in chicory (Cichorium intybus) hairy roots. New Phytol. 2009;184(1):127–40. 10.1111/j.1469-8137.2009.02924.x. 19563442

[8] Van Arkel J, Vergauwen R, Sévenier R, Hakkert JC, van Laere A, Bouwmeester HJ, Koops AJ, van der Meer IM. Sink filling, inulin metabolizing enzymes and carbohydrate status in field grown chicory (*Cichorium intybus L*). J Plant Physiol. 2012;169(15):1520–9. 10.1016/j.jplph.2012.06.005. 22795678

[9] Sakuma Y, Liu Q, Dubouzet JG, Abe H, Yamaguchi-Shinozaki K, Shinozaki K. DNA-binding specificity of the ERF/AP2 domain of Arabidopsis DREBs, transcription factors involved in dehydration- and cold-inducible gene expression. Biochem Biophys Res Commun. 2002;290(3):998–1009. 10.1006/bbrc.2001.6299. 11798174

[10] Michiels A, Van Laere A, Van Den Ende W, Tucker M. Expression analysis of a chicory fructan 1-exohydrolase gene reveals complex regulation by cold. J Exp Bot. 2004;55(401):1325–33. 10.1093/jxb/erh153. 15133058

[11] Wei H, Bausewein A, Greiner S, Dauchot N, Harms K, Rausch T. CiMYB17, a stress-induced chicory R2R3-MYB transcription factor, activates promoters of genes involved in fructan synthesis and degradation. New Phytol. 2017;215(1):281–98. 10.1111/nph.14563. 28452060

[12] Wei H, Zhao H, Su T, Bausewein A, Greiner S, Harms K, Rausch T. Chicory R2R3-MYB transcription factors CiMYB5 and CiMYB3 regulate fructan 1-exohydrolase expression in response to abiotic stress and hormonal cues. J Exp Bot. 2017;68(15):4323–38. 10.1093/jxb/erx210. PMC5853547 28922763

[13] Puranik S, Sahu PP, Srivastava PS, Prasad M. NAC proteins: Regulation and role in stress tolerance. Trends Plant Sci. 2012;17(6):369–81. 10.1016/j.tplants.2012.02.004. 22445067

[14] Liang M, Chen D, Lin M, Zheng Q, Huang Z, Lin Z, Zhao G. Isolation and characterization of two DREB1 genes encoding dehydration-responsive element binding proteins in chicory (*Cichorium intybus*). Plant Growth Regul. 2014;73(1):45–55. 10.1007/s10725-013-9866-8.

[15] Yoo SD, Cho YH, Sheen J. Arabidopsis mesophyll protoplasts: A versatile cell system for transient gene expression analysis. Nat Protoc. 2007;2(7):1565–72. 10.1038/nprot.2007.199. 17585298

[16] Singh A, Upadhyay V, Panda AK. Solubilization and Refolding of Inclusion Body Proteins. In: García-Fruitós E, editor. Insoluble Proteins. Methods in Molecular Biology. Volume 1258. New York, NY: Humana; 2015. 10.1007/978-1-4939-2205-5_15. 25447870

[17] Epstein E, Bloom A. Mineral nutrition of plants: principles and perspectives. Sunderland, Massachusetts: Sinauer Associates, Inc.; 2005.

[18] Magome H, Yamaguchi S, Hanada A, Kamiya Y, Oda K. Dwarf and delayed-flowering 1, a novel Arabidopsis mutant deficient in gibberellin biosynthesis because of overexpression of a putative AP2 transcription factor. Plant J. 2004;37(5):720–9. 10.1111/j.1365-313X.2003.01998.x. 14871311

[19] Mizoi J, Shinozaki K, Yamaguchi-Shinozaki K. AP2/ERF family transcription factors in plant abiotic stress responses. Biochim et Biophys Acta - Gene Regul Mech. 2012;1819(2):86–96. 10.1016/j.bbagrm.2011.08.004. 21867785

[20] Fuchs P, Bohle F, Lichtenauer S, Ugalde JM, Feitosa Araujo E, Mansuroglu B, Ruberti C, Wagner S, Muller-Schussele SJ, Meyer AJ, Schwarzlander M. Reductive stress triggers ANAC017-mediated retrograde signaling to safeguard the endoplasmic reticulum by boosting mitochondrial respiratory capacity. Plant Cell. 2022;34(4):1375–95. 10.1093/plcell/koac017. PMC9125394 35078237

[21] Mathieu AS, Dobrev PI, Tarkowská D, Pospíšil J, Motyka V, Jacquemin G, Quinet M, Lutts S. Phytohormone profile and CiFL1 expression in young seedlings of *Cichorium intybus* L. var sativum exposed to high temperature in relation to vernalization and de-vernalization processes. Environ Exp Bot. 2020;178:104127. 10.1016/j.envexpbot.2020.104127.

[22] Hincha DK, Livingston DP, Premakumar R, Zuther E, Obel N, Cacela C, Heyer AG. Fructans from oat and rye: Composition and effects on membrane stability during drying. Biochim et Biophys Acta – Biomembr. 2007;1768(6):1611–9. 10.1016/j.bbamem.2007.03.011. 17462587

[23] Ueno K, Yokoshima S, Sasajima Y, Ishiguro Y, Yoshida M, Shiomi N, Onodera S. 2015. Two Fructan 1-Exohydrolase Isoforms Hydrolyze Fructans in Edible Burdock (*Arctium lappa*L.) during Storage at a Low Temperature. Journal of Applied Glycoscience 62, 65–72. 10.5458/jag.jag. JAG-2014_012.

[24] Zhong J. Stress-induced degradation of inulin in Cichorium intybus: Exploring the transcriptional regulation of Fructan 1-exohydrolases. Germany: University Heidelberg; 2023. https://archiv.ub.uni-heidelberg.de/volltextserver/33699/1/Dissertation%20Jiaxin%20Zhong-2.pdf.

