# Supplementary material for: One variation, two regulatory modes? Novel insights into the transcriptional regulation of 1-FEH isoforms in chicory under abiotic stress
Source: BMC Plant Biol. 2026 Nov 10;26:1928. doi: 10.1186/s12870-026-10378-z (PMC13753121; doi:10.1186/s12870-026-10378-z)
Supplement: Supplementary file 1 [file 12870_2026_10378_MOESM2_ESM.pdf]

Supplementary data

(A)

```
                                                         AP2 domain
CiDREB1A      MLASQNPKKKA----------GRKKFKETRHPVYRGVRRRDSGKWICEVREPNKKSRVWL  97
CiDREB1C      MLASRNPKKKA----------GRKKFRETRHPVYRGVRMRDNGKWVCEMREPNKKLRVWL  93
CiDREB1D      ILASRNPKKKA----------GRKKFRETRHPVYRGVRMRDNGKWVCEMREPNKKLRVWL  93
CiDREB2B      LDEKAKPARKVPAKGSKKGCMKGKGGPENSRCNFRGVRQRTWGKWVAEIREPNRGNRLWL  110
CiDREB2A      N--NQTKPRKAPAKGSKKGCMKGKGGPENSRCQYRGVRQRTWGKWVAEIREPNRGKRLWL  118
                 . .  :*.           *   *. :  :**** *  ***:.*:****:  *:**

CiDREB1A      GTYATAEMAARAHDVAVLAMRGRSACLNFADSVWRL-------SVPESSSVQDIKKAA--  148
CiDREB1C      GTHPTAIMAARAHDVAAFAFRGRSACLNFADSVWRL-------PVPKSNKVHDIQKAA--  144
CiDREB1D      GTHPTAIMAARAHDVAAFAFRGRSACLNFADSVWRL-------PVPKSSKVQDIQKAA--  144
CiDREB2B      GTFGSAVEAALAYDEAARVMYGPCARLNLPNCRSMNEYSQ---MVPNGASSCDSTTTCSH  167
CiDREB2A      GTFGSAIEAALAYDEAARAMYGSSARLNLPNCRSMNDYIDHSRSMANPASSCDSTTTCSH  178
              **. :*  ** *:* *. .: * .* **: :.             : :  .  *  .:.

CiDREB1A      AEA------------------------AESFKPTEDAAEIVESSVEIVESSEELEE-I--  181
CiDREB1C      AEA------------------------AEAFRYTDDAEEILE--------TEELPE-I--  169
CiDREB1D      AEA------------------------AEAFRYTEDEIETVE--------TEELPE-S--  169
CiDREB2B      SEDTKTGPDSQTVKDDGESNSPNESGNSPSFKVKRE----VEKDVKVKEEPVEMEE----  219
CiDREB2A      SEGSELQMGHESVDKEP--------------------------LTVVKEEPSEEREDGGF  212
              :*                                                *  *

CiDREB1A      ------VFYMDEEEIFGMPAFLASMAEGLMVSP-PQT-LVND--NCMDNI----------  221
CiDREB1C      ------QFHVDEEDIFEMPGIFASMAEGLMIVPPPQN-VGFD--NYTDDV----------  210
CiDREB1D      ------QLYVDEEDVFEMPGIFAGMAEGLMMVPLPQN-VGYV--NYVDDV----------  210
CiDREB2B      ---DFDFQNVHVDELFDMEELLELMDQKSPNAPGLKPDPGIDSGMIMNQGGFHHPAGDTS  276
CiDREB2A      CIGEGYFQTLPEDEMFDMDELLGDLDERGGFVPKFNPDDAT-------------------  253
                        :  :::* *  ::  : :     *  :
```

(B)

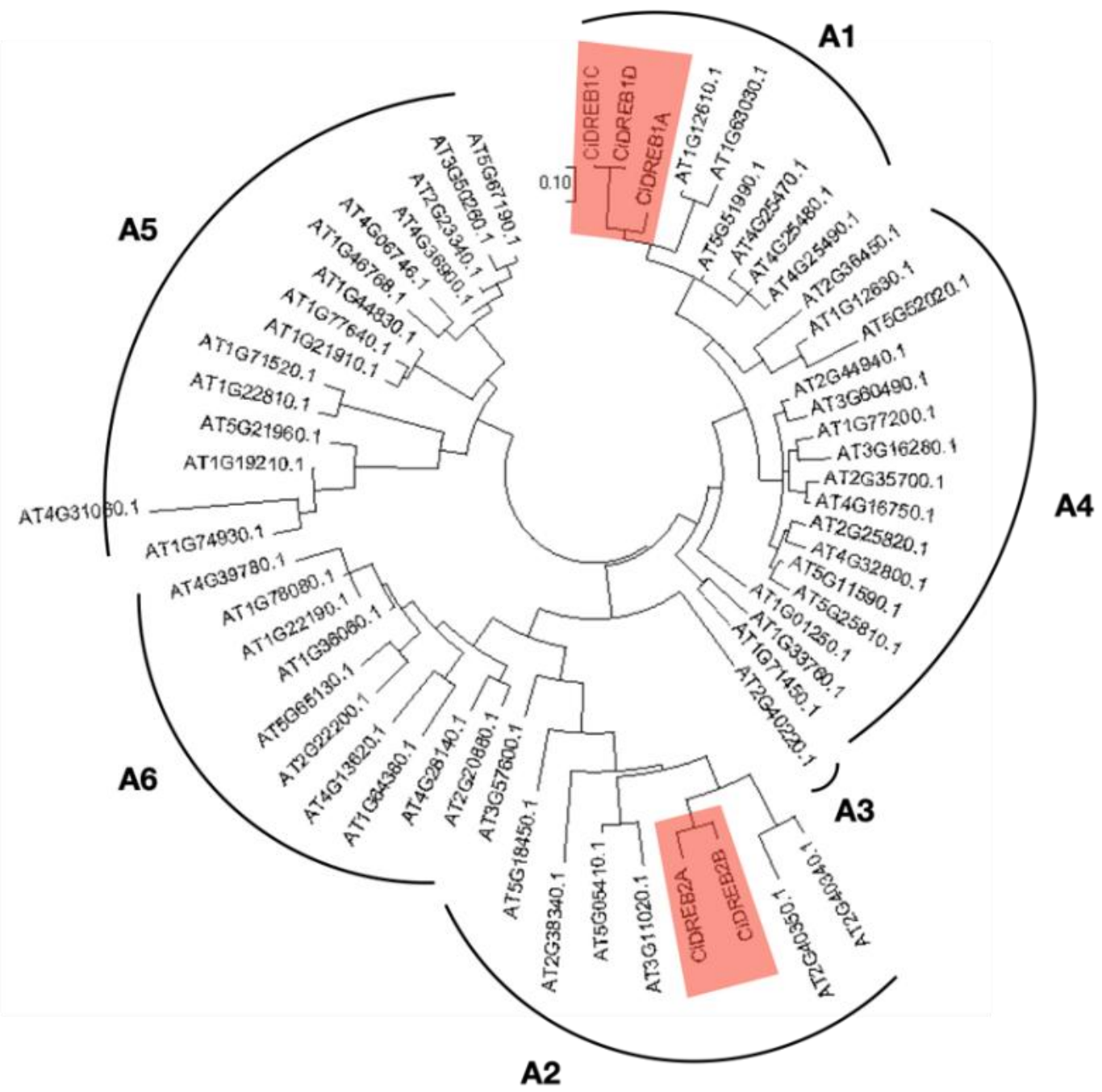

Supplementary Figure 1

(A) Comparison of the predicted amino acid sequences of CiDREB identified in chicory. Multiple amino acid sequence alignments were performed by MEGA7. The AP2 DNA-binding domain was anaylized by NCBI conserved domain search (https://www.ncbi.nlm.nih.gov) and indicated by a black line. Asterisks, colons and full stops indicate positions that have an identical, conserved and weakly conserved residue, respectively. **(B)** Molecular Phylogenetic analysis of CiDREB transcription factors in relation to AtDREB factors from *Arabidopsis thaliana* by Maximum Likelihood method. Evolutionary analyses were conducted in MEGA7.

(A)

```
CiNAC8     ------------MDVLPAKSLPVGYRFRPTDEELVNHYLRLKINGFD------KEVSCIR    42
CiNAC6     -------MEKLNFVKNGVLRLPPGFRFHPTDEELVVQYLKRKVHSCP------LPASIIP    47
CiNAC7     -------MEKLNFVNNGGLRLPPGFRFFPTDEELVLHFLKRKVQSRP------VASPIIT    47
CiNAC3     -------MGLPVTDPMTQLSLPPGFRFFPTDEELLVQYLCRKVAGHD------FSLQIIA    47
CiNAC4     -----MGVPVPVTDPMTQLRLPPGFRFYPTDEELMVQYLCRKVAGHN------FPLQIIG    49
CiNAC5     MRSPLKSVTKRIRDAESELNLPPGFRFHPTDEELIVHYLCCKSQSTAETVDAAPPPPIIA    60
CiNAC1     -------------MTGDNLVLPPGFRFHPTDEELVMHYLIKRCASQT------ISVPIIA    41
CiNAC2     -------------MTCDSLELPPGFRFHPTDDELVTHYLCRKCASQP------IVVPIIA    41
                              ** *:** ***:**: ::*  :   .            *

CiNAC8     EVDVCKKEPWDLPDLSVIESIDNEWFFFCPKDRKYQNGQRSNRATVSGYWKATGKDRTIK    102
CiNAC6     EVDVNKSDPWDLPGDL-----EQERFFFSTREVKYPNGNRSNRATASGYWKATGLDKQIV    102
CiNAC7     EADVCRSDPWDLPGDP-----KQERYFYSNMEIKYPNGKRSNRATTSGYWKATGIDKQIV    102
CiNAC3     DIDLYKFDPWELPSKAMFG--EKEWYFFSPRDRKYPNGSRPNRVAGSGYWKATGTDKVIT    105
CiNAC4     DVDLYKFDPWELPNKAMFG--EKEWYFFSPRDRKYPNGSRPNRVAGSGYWKATGTDKVII    107
CiNAC5     DVDLYKHEPWELPEMALFG--TNEWYFFTPRDRKYPNGSRPNRVTGNGYWKATGADKPIK    118
CiNAC1     EIDLYKFDPWQLPEMALYG--EKEWYFFSPRDRKYPNGSRPNRAAGTGYWKATGADKPIG    99
CiNAC2     EIDLYKFDPWQLPDMALYG--EKEWYFFSPRDRKYPNGSRPNRAAGTGYWKATGADKPIG    99
         : *: : :**:**          :* :*:    : ** **.* **.: .******* *: *

CiNAC8     TS-RGSSVIGRKKTLVFYTGRAPKGERTHWVIHEYCATEKELD----GTHPGQSPYVLCR    157
CiNAC6     TC-RTNQVVGMKKTLVFYKGKPPSGCRTDWIMHEYRLASPTLTPQPKNPTQCTENWVLCR    161
CiNAC7     DS-KTKQVAGTKKTLVFYQGKAPNGSKTNWIMHEYKLAKP---------IEGMENWVVCR    152
CiNAC3     TG---GRRVGIKKALVFYVGKAPKGNKTNWIMHEYRLSEPQRK----SGSSRLDDWVLCR    158
CiNAC4     SE---GRKVGIKKALVFYVGKAPRGSKTNWIMHEYRLSEPPKK----NNSSRLDEWVLCR    160
CiNAC5     RKSDPNSIVGIKKALVFYAGKGTKGIKTNWIMHEYRLNSTPSK--HTKNISKLDDWVLCR    176
CiNAC1     KP----KAVGIKKALVFYAGKAPRGVKTNWIMHEYRLANVDRSAGKRSNNLRLDDWVLCR    155
CiNAC2     KP----KPVGIKKALVFYAGKAPRGVKTNWIMHEYRLANVDRSAGKRNNNLRLDDWVLCR    155
                 * **:**** *:    * :*.*::***    .              . :*:**
```

(B)

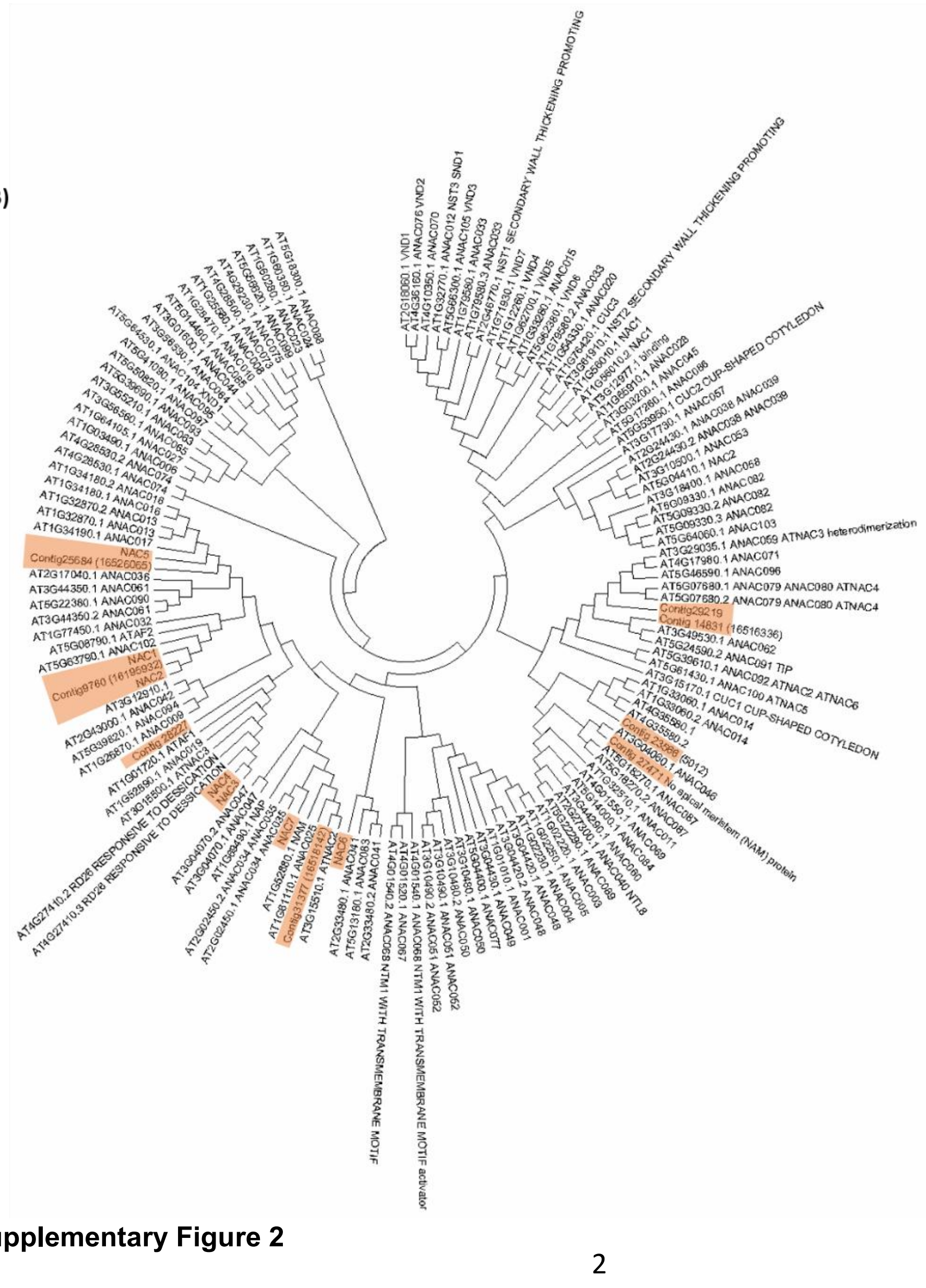

Supplementary Figure 2

(A) Multiple amino acid sequence alignments were performed by MEGA7. The NAC domain was analyzed by NCBI conserved domain search (https://www.ncbi.nlm.nih.gov/Structure/cdd/wrpsb.cgi) and indicated by a black line. Asterisks, colons and full stops indicate positions that have an identical, conserved and weakly conserved residue, respectively. **(B)** Molecular Phylogenetic analysis of CiNAC transcription factors in relation to AtNAC factors from *Arabidopsis thaliana* by Maximum Likelihood method. Evolutionary analyses were conducted in MEGA7. CiNAC identified in chicory are **highlighted** in orange.

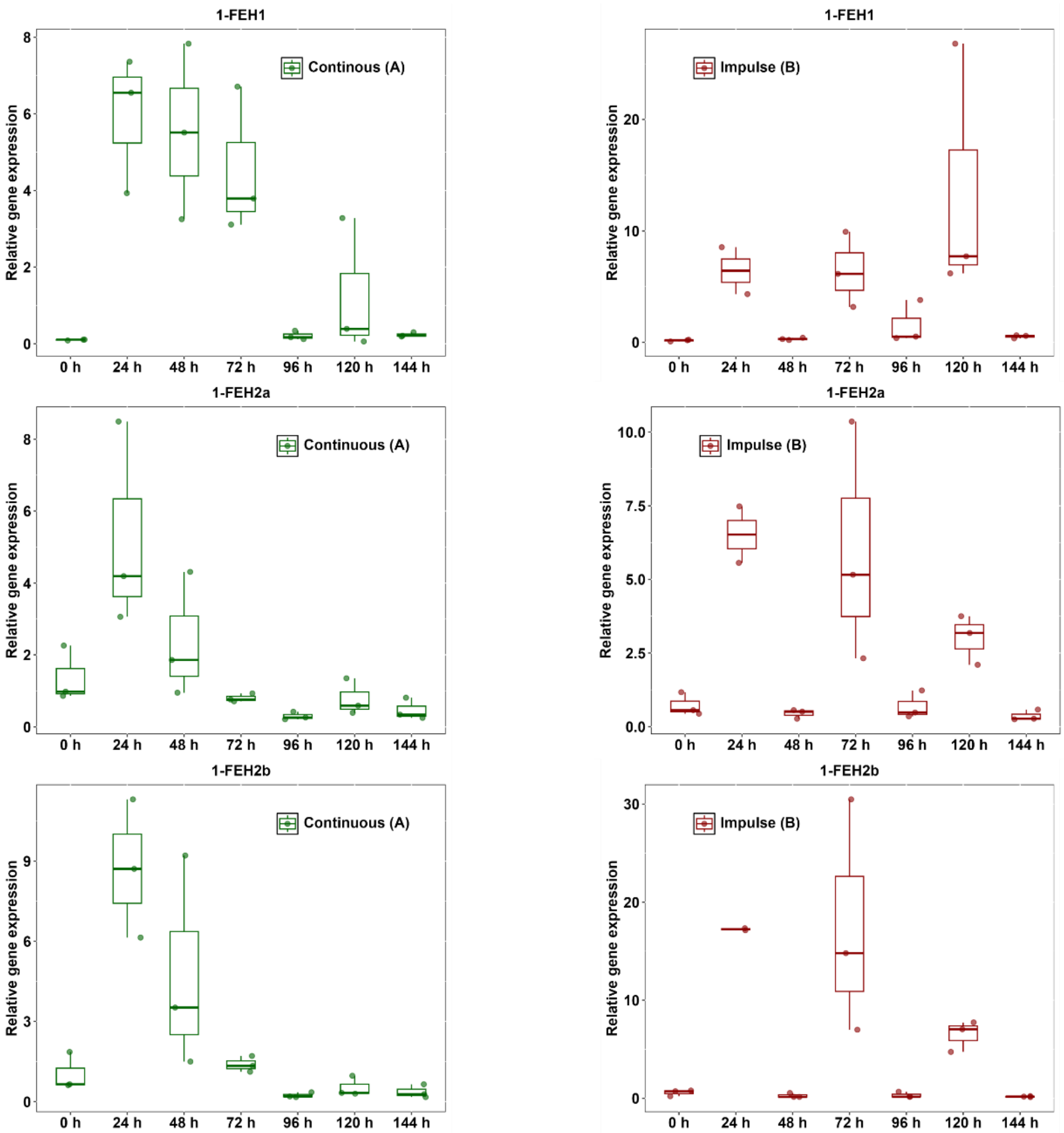

Supplementary Figure 3

Cold induction of *1-FEH* genes in 5-week-old chicory seedlings by 3 days continuous cold exposure (4 °C) followed by 3 days at RT (left panel) as compared to 3 sequential 24 h cold exposures always interrupted by 24 h at RT (right panel)

Transcript levels were determined by qPCR and normalized against the expression of two reference genes, Actin and RPL19. Relative gene expression was calculated with the delta Ct method. The error bars represent standard deviation (n = 3-4).

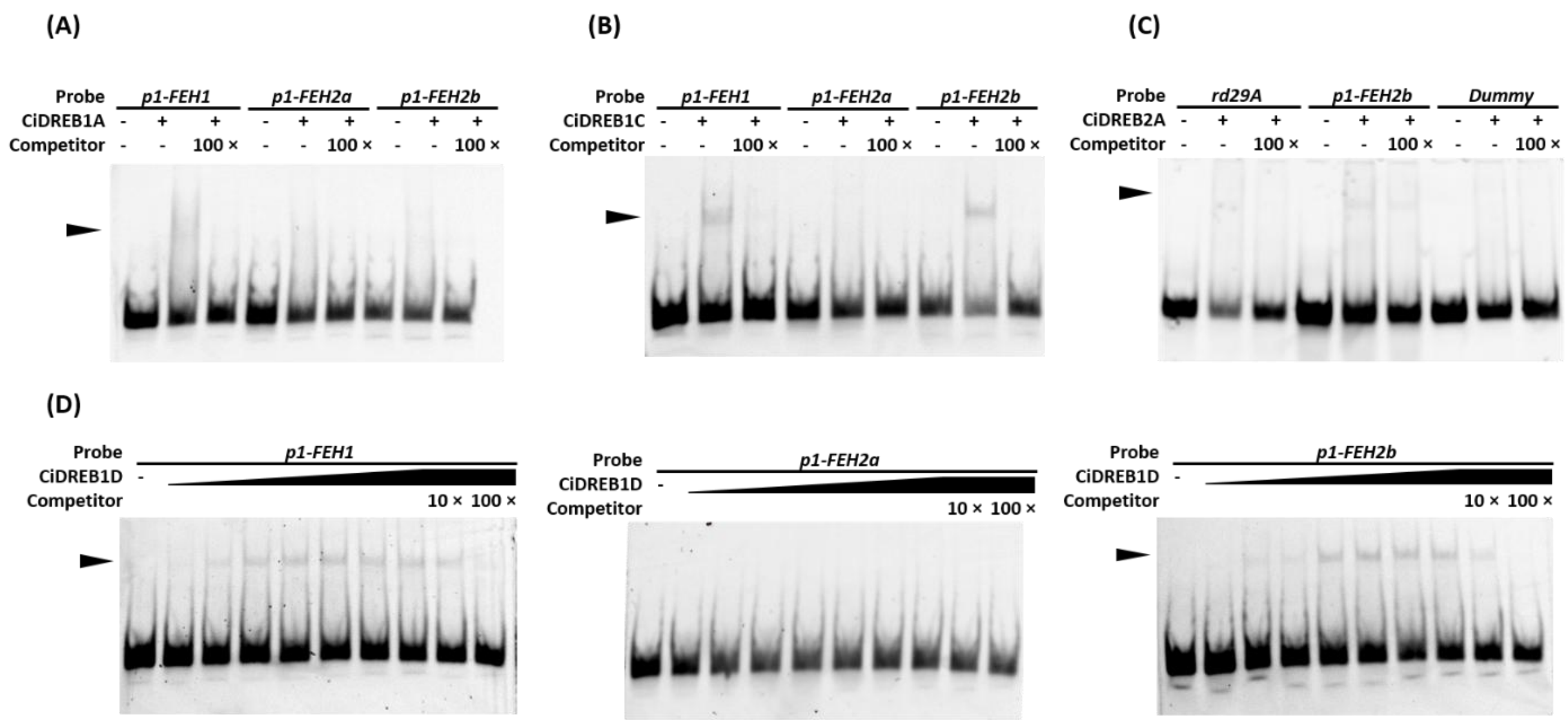

Supplementary Figure 4

Electrophoretic mobility shift assays of CiDREB proteins with promoter fragments of *1-FEH* genes

Full-length proteins of CiDREB1/CiDREB2 with 6His-tag at N-terminal was used for detecting the interaction. Black arrow heads indicate the band of DNA-protein complex. 40-mer promoter fragments were labeled with CY5 at the 5'-end. 0.2 pmol labeled probes were used for visualizing the DNA fragment. 10- or 100-times unlabeled probes were used as competitors.

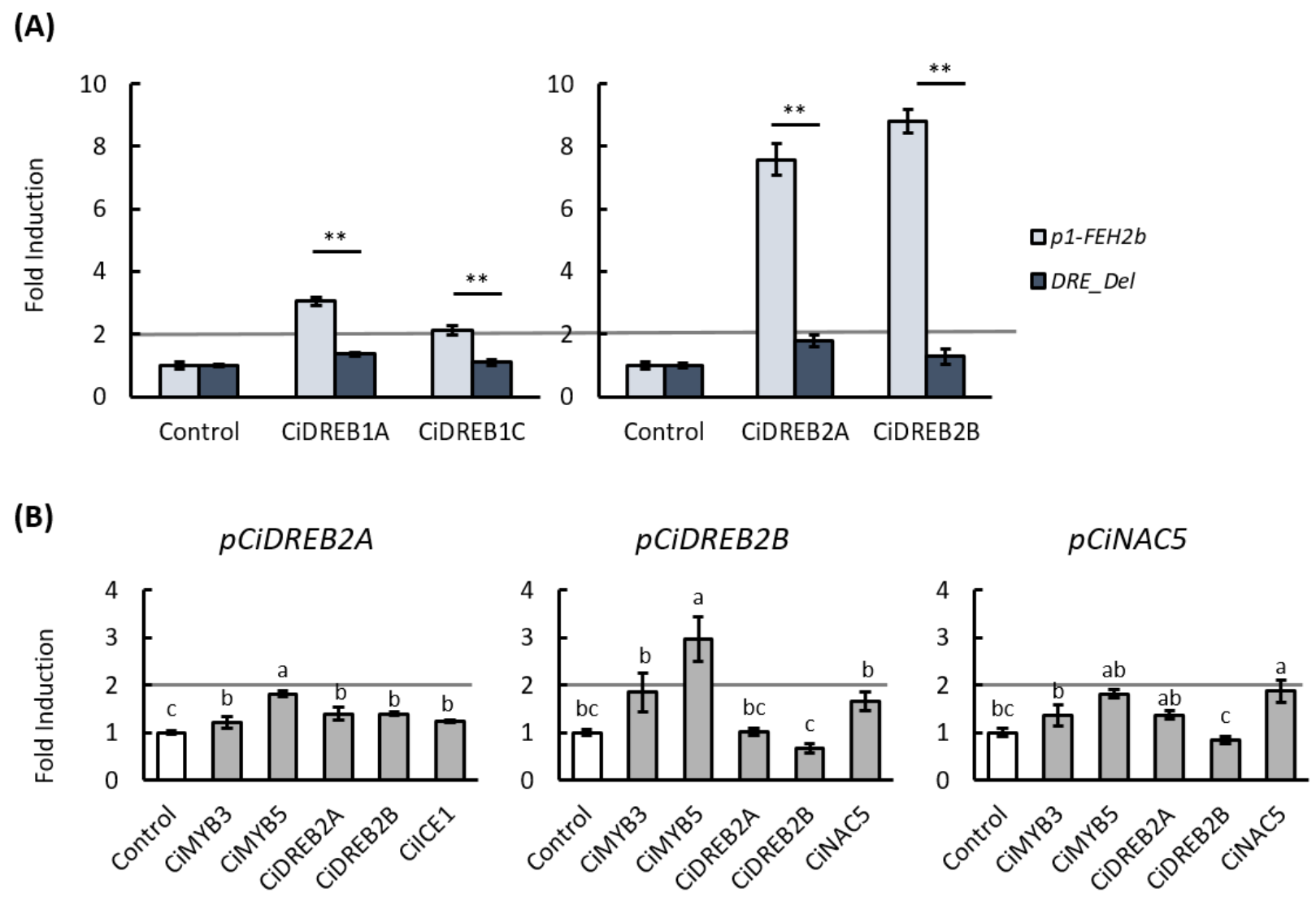

Supplementary Figure 5

In the reporter construct, partial promoter regions were fused upstream of a firefly luciferase gene. Transient expression was performed in chicory mesophyll protoplast via PEG-method. The promoter linked to a firefly luciferase reporter gene was co-transfected into cells with the effector construct (pART7-TF), or with empty pART7 vector as control. *Renilla* luciferase reporter was used as a control for normalization in each transfection. Basal promoter activity is expressed as relative LUC activity (Firefly/*Renilla*). The error bars indicate means ± SD of three technical replicates. (A) DRE_Del represents complete DRE deletion (at site -138) from *p1-FEH2b*. Fold induction of *1-FEH2b* promoter activities in the presence of CiDREBs relative to the empty vector control. (B) Effects of transcription factors (CiMYB5/3, CiDREB2A/B, CiNAC5) on their own promoter activities (*pCiDREB2A, pCiDREB2B* and *pCiNAC5*, respectively).

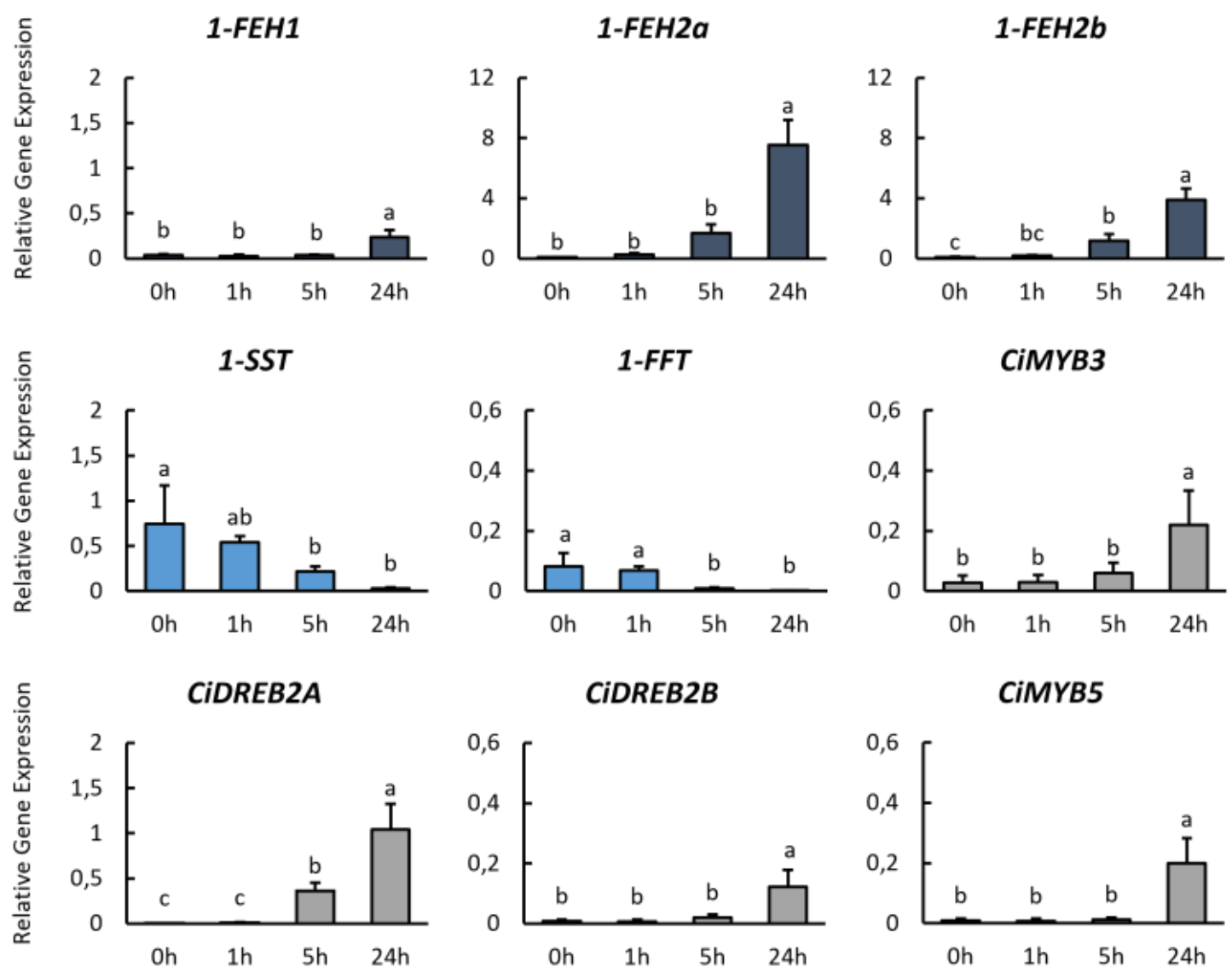

Supplementary Figure 6

Impact of heat treatment (45 °C±1) on transcript levels of FAZYs and transcription factors in taproots of 6-week-old chicory seedlings. Seedlings were transferred to incubator under 45 °C±1 and taproot samples were collected at 0, 1, 5 and 24 h after start of treatment. Transcript levels were determined by qRT-PCR and normalized against the expression of two reference genes Actin and RPL19. Relative gene expressions were calculated with delta Ct method. The error bars represent standard deviation (n = 3). One-way ANOVA test was used to determine the significances: $p \leq 0.05$.

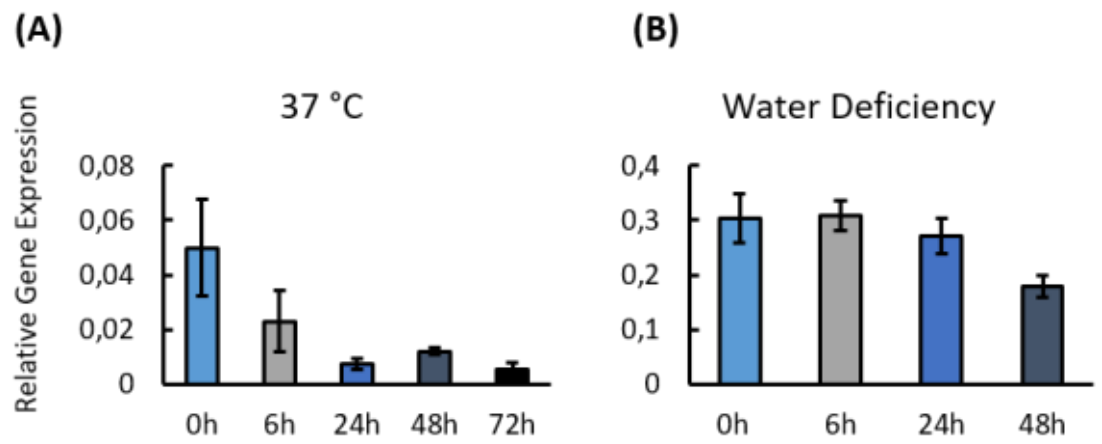

Supplementary Figure 7

Transcript levels of *CiNAC5* under heat treatment (37 °C) and water deficiency treatment in taproots of 4-week-old chicory seedling. For heat treatment, soil-grown seedlings were transferred to 37 °C for indicated time periods. For induction of water deficiency, chicory seeds were germinated on a water-soaked sponge until the first true leaves had expanded. Thereafter plants were transferred to hydroponics (Hoagland solution; Epstein and Bloom 2005). Six weeks after germination, plants were removed from Hoagland solution and their roots drained to simulate water deficiency; taproots were harvested at indicated time points.

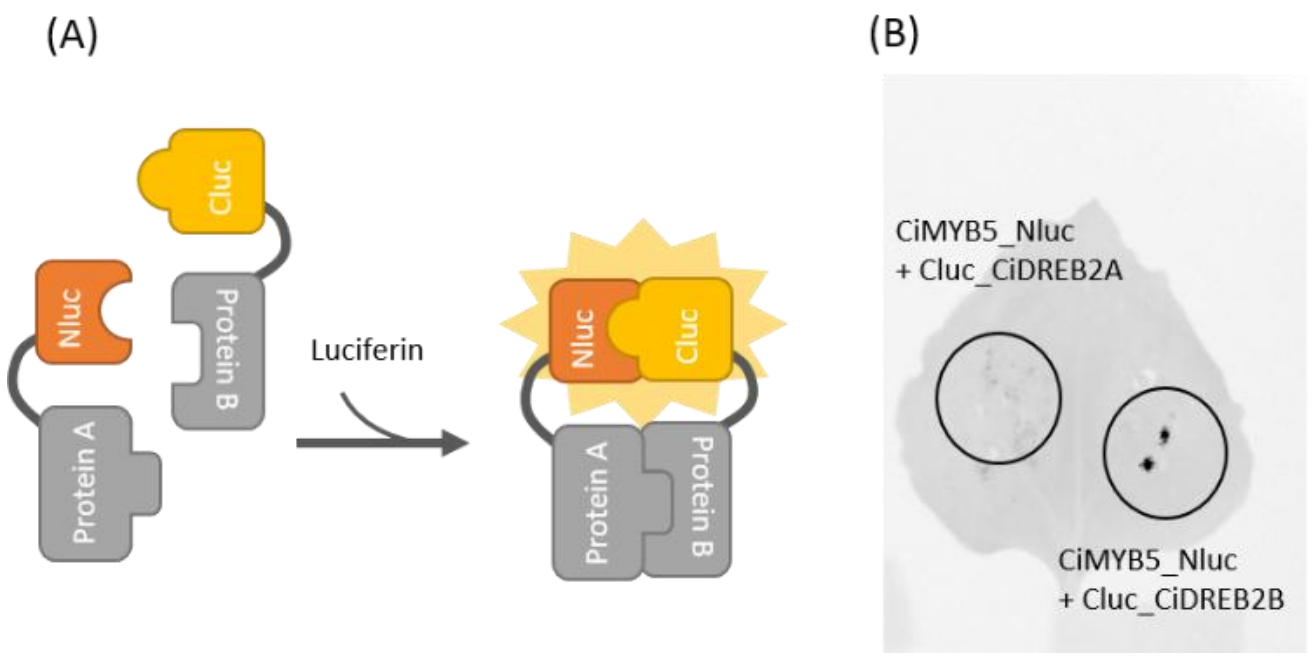

Supplementary Figure 8

Protein–protein interactions detected using a CCD imaging system. (A) Schematic representation of LUC complementation through fusion of NLuc (N-terminal domain of luciferase) and CLuc (C-terminal domain of luciferase). (B) *N. benthamiana* leaves co-infiltrated with Agrobacterium containing CLuc or CLuc and CiMYB5. Black circles indicate the regions of luminescence.

Promoter	Putative DRE element and position
p1-FEH1 (1195 bp)	ATGTCGTT (-811), ATGTCGGACG (-717), ATGTGGAC (-353), TGTGGGTG (-188)
p1-FEH2a (1147 bp)	ATGCCGTCAT (-487), TGGCCAACTT (-192), AATTCGGTAG (-136)
p1-FEH2b (1740 bp)	TGTCGGTC (-781), TGGCCAACTT (-186), AAGTCGGTAG (-138)
	**Putative NACBS element and position**
p1-FEH1 (1195 bp)	ATTACGTTAT (-1188), GTTACCTA (-1097), ACACATAA (-896), TAGGTAAC (-576), TTTCGTAC (-558), TAGGTAAC (-545), TTAGGTAAT (-517), TTTCGTAC (-500), ATAAGGTAAT (-457), ATTACATAA (-362), TACG (-279), CGTG (-167), AAACGCAA (-200), TTTCTTCTTGTG (-140)
p1-FEH2a (1147 bp)	GTTGTCGTGCTG (-1108), ATTACGTTTT (-931), ATTAATTAA (-735), GTTACATA (-674), ACAACGTAAA (-575), TTTACTTAT (-478), ACACGTAT (-228), ATAACGAAAG (-176)
p1-FEH2b (1740 bp)	GCATTTCTTGAT (-1054), TTGCTTGG (-1102), AACAAGAAAAAA (-427), TTTACTTTAC (-389)

Table S1. Prediction of DRE and NACBS on promoter sequence of *1-FEH*s

Based on the conserved binding sites of AtDREB or AtNAC from *Arabidopsis thaliana*, the promoters of *1-FEH1*, *1-FEH2a* and *1-FEH2b* were screened in JASPAR database (jaspar.genereg.net) for the presence of *cis*-elements containing the core motifs of DRE (Dehydration Response Element) or NACBS (NAC Binding Site).
