# Supplementary material for: One variation, two regulatory modes? Novel insights into the transcriptional regulation of 1-FEH isoforms in chicory under abiotic stress
Source: BMC Plant Biol. 2026 Nov 10;26:1928. doi: 10.1186/s12870-026-10378-z (PMC13753121; doi:10.1186/s12870-026-10378-z)
Supplement: Supplementary file 2 [file 12870_2026_10378_MOESM1_ESM.docx]

Promoter sequences

>p1-FEH1

TTACCGGTAGATTACGTTATTATGAACAGGTATAACAAGTATACTGGTCAAATGTATACAAAAAAACATTCTTACCTAAACGTGGACAAAAACCAAAGTTCGTTACCTATTTATTATACATCTAAAATGTCCCAATTTTTTATGTTTAATTATTGTATTTTAATTTTTTTAGAAATTAACCCCATGATCGGTTTTTACATCATTTTCGAATATATGAATAATTAGATGCTTTACAACTAAGTACCATTTAAAACCTGAAAATGACTTATTTTGAAAAGAGTTAAAAATTGTAATGGTCTTAAACACATAAAAACGATTATAGGCAAAAAGATTAAAAAATAATGAAATAATATTACATTCATCTTGTTCACTGCTTTATCCTAACTCATGTCGTTTTAAATGAACAATCGAGTCTTCGAGGTAAAAATGGTGAACAATGAGTCTCTTGAAGTTGTATGAAACGATAAATTCATTGGCCTTAATGTCGGACGAAATTATATATCTTTTAATTTGTTATTTCAAATTAAAAGCACTAAGATCTAAAAACTATTATATTAGAATACAGAAGTATTTAAAAGCATAAAACAATTAAAGAGTTTTCCTTCATGCATCCGTCCAAATTTAGGTAACCATATTTTTTTTTCGTACATGTTTAGGTAACCATGTTTTTTTCATACTTGTTTAGGTAATAATGTTTTTTTCGTACATATTTGACCATTATACTTGTTAGGATTGTTCATAATAAGGTAATATTACTGGTAATCGTAGATAATGTTACTTTGTTATGAACAGGTCAAACAAGTATACTGGTCAAATATGTAAAAAAAAAAAACATGATTACATAAATGTGGACAAACACCGAACCTCATTACCCATTTATTTCATTAACCCTTTGTTTTATGCTAATTGCTAAACTTGGTACGCCTTATTTTTTATGCTTATGAGTGTGACCACCAAAGTATTTGACAACGACTGTTTCCTTTTTCCAATATAAGATGAAACGCAATCATTGTGGGTGAGACTAGTCCATCCGTGGAGGGAGTAATCAAAATTCGTTTTTTCTTCTTGTGTATTCACTATAAATACTAGCCATGCATATGCCAAAGCTAAACAACACTAGAACCACATAATTCTCGATGTTCTTTTTCTTTTCACGATCATTGGTGAGTTAATAAACCTGTCAGATCGACACTGAACAATG

>p1-FEH2a

CCGCAGACCTCTATCCATATATTAGTTCTTTTTCTCCATATGTTGTCGTGCTGTCCAAACAGTCACTTTTCGCTTTTTGAATAGTTATATCTTTTATCATTGCAATGAATGATATTTTTGATCAATAATATCAATTATCTGTATTAAATAGATATTTTTAGTTTTATATGCTGTATAATTACTATGTTTGTCCAATAATGCTAGCTTTTACATCTCCAATTACGTTTTTACCATCAAAGAATTATTATTTTTTGATTTCAGATTTTGTAGTATATATTTTAATTTCGTCTATGGTAATATATTGATTTTATTTAATGTTATTGTTAACTAGTTTTTAATCTTATATATTCTAGATGTCCTTAAAAATAAAAAATTAAATGATGTTTTATAAAATATTTCATAAATATGAGGACAATTAATTAACTTAAACAACAAATTAGTGGTCAATGGTTTGTTTAATAAATTTAGTGGAAATGTTACATAGAAAAAACATGGCTCATGAGCCCTGTGATCGTGAATGTTCTATTACTTTTCTATTCAGAACGTGCAACATGGACTAGTAGTTTCTTTCAAAACAACGTAAAAGTATAAAACTATTGATTTATCCATAAATAAAAATTGCGCTCGTATATGTAAAAGAAACTACAAACTCATAAATGAATATGCCGTCATTTACTTATTAATACTTATAAATGTACTGGTAATGAGATAGAAGTAAAATGTTTATCAAATGTCAATAGAGAAAAACTCTATACAACTCAAACTAAAACGGATGTATTTTTAAATAGACCATATTATACATCTTTACAAAGAAAAACATAATTTAGAAGTTAGTCCTTTGTTACGGCGGCTAGATATTTCAAAGTCGTAAAAAATGGGTCCTTTAAAGACAATGAAATAAAAAATACATAATCGAGGATGACACGTATATTAATAGTAAAAAGTGAGGGGTAGGCCTGGCCAACTTTTCAAAATAACGAAAGTTCAAGGGGCAATAAGTCAATAACTAACATAATTCGGTAGATACTATTTGTCTCAAAGTCTATAAAACCAACCAATGGACCATTCTCAATTCTCATTCCATCCATAACAACACCCATTCTCCTCAACAACATTAAAGTCATCGATCCCCAAACACACACACTCATCATG

>p1-FEH2b

GGGGACAAGTTTGTACAAAAAAGCAGGCTCTTAACAGAATGGACCCAATTATTCGATTATCACTATATGGGTCACTAAGGTTTGCAAAATACAACAAATCGGTCTATGAGTAAAGTAAAAAGACTAAAATATCATTTTAAATTTCTTTTTTCTTTTTCTTTGAATTTATTATTTATTTTATTAATAAAAAAAGAAAATACTCCAACCCACCCCCACCCCCACCCCCACGTTTCTCTCTATCTCTCTCTCTCTCTCTCTCTCCTCTGCGTACATAACAAAAAGGGCAGCAGCCCATCTTTACCCCCATGATCACAGTGGCGCTTTTCATCCTTGAATTGAGCCATTTAGCAACTCAATCTCGGAATGCTTTCAATCATATCTGTTAAGTTATCCATTGATGTAGTTCTAGCCATGAAACGATAGACTTTAGTTAGTTATTTAGGTTTATTTAGCCAGAAATAGTCAAGGAAGCCATTGAAGTTGCAGGTTTTCACATCGCTGGATTTCCAGATCAAGACATAGCAGTATGCCGCCTTAAAATATAGGGAATGACATGCACAAGTTGCCAAGAAAACCAGATCATGAGGATTATGGTGTAGATTAAGGAATAAAAAACAAAGGAGCTCTCAAAAGTCAGAAAATTCGTTATCTCAGTTCTCCCTTGGTCCTTGCTTGGTCCTTGCTGGGATTGTGTTTTTTTTCATATGGCGCCTCAAGCATTTCTTGATTGCTGTAATTTGTAATATGGTTGCTTTTGCGAATTTGCAAAATTATAAACGTATTATCAAATCGTTGCAGATTATCTAGAGATAGATGATGAAGCAAGATTAATGGCGGAAGAATTGGGAACCGATCGAATCTTGATCGATCTAGGGTATCGAATAATTAGCGATTACAACAAGGAGAATGTACGATCGGCTCTAGATAACAATGAAACCGCACACAGTAACCAGGATTGTATCGTTGGATTGGGGCTCGATATTTCTACAGTGTCGGTCTTAGTCGCTCCCCGATTTATGCAATCGGACATGTTTCTCCATCCAATGATTCAACAAAATCGAAGACATCCGGCAAGCAGAAGAATATCATCATGGCTGGCAAATGGTGTGGGAAAGATTGGATGTCAAATCAAGTTCATCAATTTCTAGTTGTTGAATCGTATTGAGAGAGAGAGAGAGAGAGAGAGAGAGAGAGAGAGAGAGAGAGAGAGAGAGAGAGACGTGGAGGTGGAGAGAGAGAGAGAGAGAGAGAGAGAGAGAGAGAGAGAGAGACGTGGAGGTGGGGGTCGGGGTGAGGTATTTTCTTTTTTATTATTATTAATAAAATAAATAATAAATTCAAAAAACAAGAAAAAAGAAATTAAAAATGATATTTAAGTATTTTTACTTTACTCAGGGACCGATTTGTAGGATTTTGCAAACCTTAGTGACCTATATAGTGAAAATCAAATAGTTGGGACCATTTGTTAGTTTTGGTAAACCACAGGGACCAAAACTGTAATTTACTCGTCCTTTAAAGACAATGAAATATAAAATGCATAATCGAGGATGACGCGTATATTATACTAAAAAGTGAGGGTAGGCCTGGCCAACTTTTCAAAATAAAGAAACTTCAAGGGGCAATAACTAACATAAGTCGGTAGATACTATTTGTCTCAAAGTCTATAAAAACAACCAATGGACGATTCTCAATTTTCATTCCATCCATAACACCCATTCTCCTCAGCCCAGCTTTCTTGTACAAAGTGGTCCCC

Coding Sequences

>CiDREB1A

ATGAATATCTTTATCGAATCCCATATCCCATTTCCTGAACTATCATCGGAAAATACTCCGGTTGAGTCCTCTTCCTCTTCTGATTGCAGCAGCAGCACCACCACCACCGTCGGAGGTGCTTATTATGCGGAAGCGGAAGTGATGCTGGCTTCACAAAACCCCAAGAAGAAAGCTGGAAGGAAGAAGTTTAAGGAGACTCGACACCCAGTTTACCGGGGAGTGAGGAGGAGGGATTCCGGTAAGTGGATTTGTGAAGTGAGAGAGCCTAACAAGAAGTCGAGAGTGTGGCTGGGAACGTATGCGACGGCTGAAATGGCAGCCAGGGCACATGACGTGGCGGTTTTGGCCATGAGGGGGCGATCGGCGTGTTTAAATTTTGCTGACTCAGTGTGGAGGCTGTCAGTGCCGGAGTCTAGCAGTGTACAGGATATAAAGAAGGCGGCGGCTGAGGCGGCGGAAGCTTTTAAACAGACGGAGGATGCGGCGGAGATTGTGGAAAGTTCGGTGGAGATTGTGGAAAGTTCGGAGGAGTTGGAAGAAATCGTGTTTTACATGGATGAAGAGGAGATATTTGGGATGCCGGCATTTCTTGCCAGCATGGCGGAGGGACTGATGGTGTCGCCACCTCAGACGCTAGTGAACGACAACTGTATGGATAACATTGAATTCTGTGTTGACTTACCTTTATGGAGTTTCTAG

>CiDREB1C

ATGGATACCTTTATCGAAACATATAACCCATTTCCAGCACTATCATCGGAAGATGTTCCGGTAGCATACTTCTTGACCTCAAATTGCAGTAGAACCACCGTCCTGAATGCGGCTGCTGAAGCAGAAGTGATGTTGGCTTCACGAAACCCGAAGAAGAAGGCCGGAAGGAAGAAGTTCAGGGAGACTCGACACCCAGTTTACCGGGGAGTGAGAATGAGGGATAACGGTAAGTGGGTTTGTGAGATGAGAGAGCCGAACAAGAAGCTGAGAGTGTGGCTCGGGACGCATCCTACCGCGATAATGGCAGCCAGGGCACATGATGTGGCGGCGTTCGCCTTCAGGGGACGATCGGCGTGTTTGAATTTTGCTGACTCAGTGTGGCGGTTGCCGGTGCCGAAGTCTAACAAAGTGCATGATATACAGAAGGCGGCAGCAGAAGCGGCGGAGGCTTTCAGATACACGGACGATGCGGAGGAGATTTTGGAAACGGAGGAGTTGCCGGAAATTCAGTTTCATGTTGATGAAGAGGATATTTTCGAGATGCCCGGAATTTTCGCCAGCATGGCGGAGGGACTGATGATTGTACCACCTCCTCAGAATGTGGGGTTTGACAACTACACGGATGACGTGGAATTTTGTGCTGAAGAGTCTTTATGGAGCTTTTAG

>CiDREB1D

ATGGATACCTTTATCGAAACATATAACCCATTTCCAGCACGATCATCGGAAGATGTTCCGTTGGCATACTTTTTGACCTCAAATTGCAGCAGAAGCACCCTCCTGAATGCGGCTGTTGAAGCAGAAGTGATATTGGCTTCACGGAACCCGAAGAAGAAGGCCGGAAGGAAGAAGTTCCGGGAGACTCGACACCCGGTTTACCGGGGAGTCAGGATGAGGGATAACGGTAAGTGGGTTTGTGAGATGAGAGAGCCGAATAAGAAGCTGAGGGTGTGGCTCGGAACGCATCCTACTGCGATAATGGCGGCCAGGGCACATGATGTGGCGGCGTTCGCCTTCAGGGGACGATCGGCTTGTTTGAATTTTGCTGACTCAGTATGGCGGTTGCCGGTGCCGAAGTCTAGCAAAGTACAAGATATACAAAAGGCGGCGGCAGAAGCGGCGGAGGCTTTTAGATACACGGAGGATGAGATTGAGACTGTGGAAACGGAGGAGTTGCCGGAAAGTCAACTATACGTGGATGAAGAAGATGTTTTCGAGATGCCCGGAATTTTTGCCGGTATGGCAGAGGGACTGATGATGGTTCCACTTCCTCAGAATGTAGGGTATGTCAACTATGTGGATGACGTGGAATTTTGTGCTGAAGAGTCTTTATGGAGCTTTTAG

>CiDREB2A

ATGTCGTCGGGGTTTACTGATAGAAACACTATGATGGATCTTCTTAGTCAAACTTCCAGTACACTTTATACACCAATGGATTCCTCAAGAAAGAAGATAACAAGAAGTAGACGTGATGCACCAAAGAAAGTGGCTGCAACTCTTGCAAAGTGGATAGAATACAACAACATGGACTCACCAAATAACCAAACAAAACCCCGAAAAGCCCCTGCTAAAGGATCCAAGAAAGGTTGCATGAAGGGTAAAGGAGGACCTGAAAACTCCAGGTGTCAGTATAGAGGAGTAAGGCAAAGAACATGGGGTAAATGGGTAGCAGAGATTCGGGAACCCAACAGGGGTAAACGTTTATGGCTTGGAACTTTTGGGTCAGCCATTGAAGCTGCCCTAGCTTACGATGAAGCTGCCCGAGCAATGTACGGCTCTTCCGCTCGTCTCAATCTTCCAAATTGTCGGTCAATGAATGATTACATTGACCATTCGCGGTCAATGGCTAACCCTGCTTCTAGTTGTGACTCTACCACCACTTGCAGTCACTCTGAGGGCAGTGAGCTGCAAATGGGACATGAATCTGTGGATAAAGAACCGTTGACTGTGGTCAAAGAAGAACCTTCCGAGGAGAGAGAAGATGGGGGATTTTGTATTGGTGAAGGTTACTTTCAGACGTTACCTGAAGATGAGATGTTTGATATGGACGAGCTTCTTGGGGATTTGGATGAAAGAGGTGGTTTTGTACCCAAATTTAACCCAGATGATGCTACTTATGAGCTGGACAATCAAAACACGTGGCAAGATCCTAGTGGGGGTGTTGATTATAGTTTGGAGTTCTTGAAGCCGGGAAGGCCTGAAGATGATAGTTTCACTTTGGAAGAGTTGGATCTGAGTTTTGATCAGGAGTTAGGTGTGTAA

>CiDREB2B

ATGTTCATCAACAGAAATACATCCAACACAGTCCTCCCTTCATCAATGGATTCATCCAGGAAGAGAAAATCACGAAGTAGACGTGACGGCCCAAAAGGAGTAGCCGAAACACTCGCCAAATGGAAGGAATACAACAACAAGATCGAGAAATTGGATGAAAAAGCAAAACCAGCACGTAAAGTTCCCGCAAAAGGGTCGAAGAAAGGTTGCATGAAAGGTAAAGGAGGACCTGAAAACTCCAGGTGTAACTTTAGAGGCGTAAGACAACGAACATGGGGTAAATGGGTCGCTGAAATTCGAGAACCCAACAGAGGGAATCGTTTATGGCTTGGAACGTTCGGGTCAGCCGTCGAAGCTGCCCTAGCTTACGATGAAGCTGCACGAGTCATGTACGGTCCTTGTGCTCGTCTCAATCTACCAAACTGTCGATCCATGAACGAGTATTCACAAATGGTGCCTAATGGCGCGTCTAGTTGCGACTCGACGACAACATGTAGTCATTCCGAAGACACTAAAACCGGTCCAGATTCACAAACGGTCAAGGACGACGGCGAATCAAATAGCCCAAACGAATCCGGTAATTCACCGTCGTTTAAAGTGAAACGAGAAGTTGAAAAAGATGTTAAAGTGAAGGAAGAACCTGTAGAAATGGAGGAAGACTTCGATTTCCAAAACGTACATGTCGACGAACTGTTCGATATGGAGGAGCTTCTCGAACTGATGGACCAAAAAAGCCCTAACGCACCTGGTTTAAAGCCCGACCCGGGGATCGATTCAGGGATGATCATGAATCAAGGCGGATTCCACCACCCTGCCGGCGATACTTCTGAGCAATGGCGTGATGGAAGTAACCGGAGTTCACCTTACCGGCAACAAGAAACTTGCCGGAATGCTACTGGTACGCCGGAGCTTGACAATAAGCCTTGGAGCCTTGATAACCGGAACGAACAGGAAGATGGATCTGATTACGGGTTTGATTTTTTGATGCCCGGTCGACCTGAAGATTATAATTTGACGTTAGATGATTTAGGGTTTGATTTTGGAGGTGATTTGGGTGTGTAA

>CiNAC5

ATGAGATCGCCGTTGAAATCCGTAACAAAAAGGATAAGAGATGCTGAATCTGAGTTGAATTTGCCTCCCGGCTTCCGATTTCACCCAACGGATGAGGAGCTCATCGTTCACTATCTCTGCTGCAAGTCCCAATCGACGGCGGAAACCGTTGATGCCGCTCCACCACCACCGATCATCGCTGATGTTGATCTCTACAAACACGAACCATGGGAGCTTCCTGAAATGGCTCTGTTTGGAACAAACGAATGGTACTTCTTCACACCAAGAGACAGGAAATACCCTAACGGGTCAAGACCAAACCGAGTGACAGGAAACGGGTACTGGAAGGCCACCGGAGCCGATAAGCCCATAAAGAGAAAATCCGATCCGAATTCAATCGTTGGAATTAAGAAAGCCTTGGTTTTTTACGCGGGAAAAGGGACTAAAGGGATCAAGACCAATTGGATCATGCATGAATATAGACTAAATTCTACCCCTTCAAAACACACCAAAAACATCTCCAAGTTGGACGATTGGGTTTTGTGTCGTCTTTACAACAAGAAGAACAATCCGAACGAGAAGATGATACCAGAAGATAATAACATCGACCATAGCCGTGATCTTCATCCTACATCACCATTAGATGAATCAGAAAGAGGAAATAGTTGTAGTAACAACAGTCACAGTCTTAATACATTTGAAGATTCAGATGGTGAATTTGGTGGTAACTTTGAAGGTGACGTCATGTTCATAAGCGACTTGCCGCGTGAAGTTTTGACCAAAGTCAAAGGTACAAGTGAGCAAAGCAGCATCGACCTAGTGGAAAATTTGATTCAAGATGGGAATGAGTGGCTGGACAGTATCAGTTTGGAAGATATGCATCATTTTCTGGAGTCAATGCCTCCTAATCATGACATTTATGAAATGCCACTGATTTATAACCAAGATTTTGATTATTTGATCCATGGAGTTTAA

Protein sequences

>CiDREB1A_1

MNIFIESHIPFPELSSENTPVESSSSSDCSSSTTTTVGGAYYAEAEVMLASQNPKKKAGRKKFKETRHPVYRGVRRRDSGKWICEVREPNKKSRVWLGTYATAEMAARAHDVAVLAMRGRSACLNFADSVWRLSVPESSSVQDIKKAAAEAAEAFKQTEDAAEIVESSVEIVESSEELEEIVFYMDEEEIFGMPAFLASMAEGLMVSPPQTLVNDNCMDNIEFCVDLPLWSF*

>CiDREB1C_1

MDTFIETYNPFPALSSEDVPVAYFLTSNCSRTTVLNAAAEAEVMLASRNPKKKAGRKKFRETRHPVYRGVRMRDNGKWVCEMREPNKKLRVWLGTHPTAIMAARAHDVAAFAFRGRSACLNFADSVWRLPVPKSNKVHDIQKAAAEAAEAFRYTDDAEEILETEELPEIQFHVDEEDIFEMPGIFASMAEGLMIVPPPQNVGFDNYTDDVEFCAEESLWSF*

>CiDREB1D_1

MDTFIETYNPFPARSSEDVPLAYFLTSNCSRSTLLNAAVEAEVILASRNPKKKAGRKKFRETRHPVYRGVRMRDNGKWVCEMREPNKKLRVWLGTHPTAIMAARAHDVAAFAFRGRSACLNFADSVWRLPVPKSSKVQDIQKAAAEAAEAFRYTEDEIETVETEELPESQLYVDEEDVFEMPGIFAGMAEGLMMVPLPQNVGYVNYVDDVEFCAEESLWSF*

>CiDREB2A_1

MSSGFTDRNTMMDLLSQTSSTLYTPMDSSRKKITRSRRDAPKKVAATLAKWIEYNNMDSPNNQTKPRKAPAKGSKKGCMKGKGGPENSRCQYRGVRQRTWGKWVAEIREPNRGKRLWLGTFGSAIEAALAYDEAARAMYGSSARLNLPNCRSMNDYIDHSRSMANPASSCDSTTTCSHSEGSELQMGHESVDKEPLTVVKEEPSEEREDGGFCIGEGYFQTLPEDEMFDMDELLGDLDERGGFVPKFNPDDATYELDNQNTWQDPSGGVDYSLEFLKPGRPEDDSFTLEELDLSFDQELGV*

>CiDREB2B_1

MFINRNTSNTVLPSSMDSSRKRKSRSRRDGPKGVAETLAKWKEYNNKIEKLDEKAKPARKVPAKGSKKGCMKGKGGPENSRCNFRGVRQRTWGKWVAEIREPNRGNRLWLGTFGSAVEAALAYDEAARVMYGPCARLNLPNCRSMNEYSQMVPNGASSCDSTTTCSHSEDTKTGPDSQTVKDDGESNSPNESGNSPSFKVKREVEKDVKVKEEPVEMEEDFDFQNVHVDELFDMEELLELMDQKSPNAPGLKPDPGIDSGMIMNQGGFHHPAGDTSEQWRDGSNRSSPYRQQETCRNATGTPELDNKPWSLDNRNEQEDGSDYGFDFLMPGRPEDYNLTLDDLGFDFGGDLGV*

>CiNAC5_1

MRSPLKSVTKRIRDAESELNLPPGFRFHPTDEELIVHYLCCKSQSTAETVDAAPPPPIIADVDLYKHEPWELPEMALFGTNEWYFFTPRDRKYPNGSRPNRVTGNGYWKATGADKPIKRKSDPNSIVGIKKALVFYAGKGTKGIKTNWIMHEYRLNSTPSKHTKNISKLDDWVLCRLYNKKNNPNEKMIPEDNNIDHSRDLHPTSPLDESERGNSCSNNSHSLNTFEDSDGEFGGNFEGDVMFISDLPREVLTKVKGTSEQSSIDLVENLIQDGNEWLDSISLEDMHHFLESMPPNHDIYEMPLIYNQDFDYLIHGV*
